# Effect of Skeletal Muscle Mass and Its Associated Mediators on the Development of Steatotic Liver Disease: A Cohort Study in China

**DOI:** 10.1002/jcsm.70093

**Published:** 2025-11-04

**Authors:** Yebei Liang, Rong Yu, Xiaoqi Ye, Lu Yang, Fusong Jiang, Liang Feng, Lichang Zhong, Keqing Dong, Li Wei, Yuqian Bao, Xuhong Hou, Weiping Jia

**Affiliations:** ^1^ Shanghai Diabetes Institute, Shanghai Key Laboratory of Diabetes Mellitus, Department of Endocrinology and Metabolism, Shanghai Clinical Center for Diabetes, Shanghai Key Clinical Center for Metabolic Disease Shanghai Sixth People's Hospital Affiliated to Shanghai Jiao Tong University School of Medicine Shanghai China; ^2^ Department of Geriatrics The First Affiliated Hospital of Fujian Medical University Fuzhou Fujian China; ^3^ Department of Endocrinology and Metabolism West China Hospital, Sichuan University Chengdu Sichuan China; ^4^ Department of Specialized Medicine Shanghai Sixth People's Hospital Affiliated to Shanghai Jiao Tong University School of Medicine Shanghai China; ^5^ Department of Ultrasound in Medicine Shanghai Sixth People's Hospital Affiliated to Shanghai Jiao Tong University School of Medicine Shanghai China; ^6^ General Practitioner Teams in Community Health Service Center of Nicheng, Pudong New District Shanghai China

**Keywords:** appendicular skeletal muscle mass, community‐based prospective cohort study, mediators, metabolic dysfunction and alcohol‐associated liver disease, metabolic dysfunction–associated steatotic liver disease

## Abstract

**Background:**

Understanding the relationship between relative skeletal muscle mass and newly proposed steatotic liver disease (SLD) is crucial, but research gaps still exist. Based on a cohort study, we investigated the impact of relative skeletal muscle mass on incident SLD and its subtypes and explored potential mediators involved in these relationships.

**Methods:**

We followed 1964 subjects aged 55–70 years (median age: 61.4 [58.4–65.1] years; 45.5% male participants). Appendicular skeletal muscle mass (ASM) was measured using bioelectrical impedance analysis and adjusted for height squared (ASM/height^2^), weight (ASM/weight) and body mass index (ASM/BMI) to quantify relative skeletal muscle mass. SLD was diagnosed using ultrasonography and classified into metabolic dysfunction–associated steatotic liver disease (MASLD), metabolic dysfunction and alcohol‐associated liver disease (MetALD) and alcohol‐associated liver disease (ALD).

**Results:**

Over a mean 4.3‐year follow‐up period, 598 participants (30.4%) developed SLD: 539 MASLD (27.4%), 38 MetALD (1.9%) and 21 ALD (1.1%). Higher ASM/weight and ASM/BMI were associated with lower risks of SLD and MASLD (RR per SD [95% CI]: 0.71 [0.61–0.82], 0.59 [0.46–0.76]; 0.66 [0.58–0.76], 0.51 [0.40–0.64]; all *p* < 0.001). Associations of ASM/height^2^ with SLD and MASLD shifted from positive to negative after adjustment for BMI (from 1.67 [1.51–1.84] to 0.77 [0.67–0.89]; from 2.30 [1.92–2.76] to 0.61 [0.47–0.79], all *p* < 0.001). ASM/height^2^ and ASM/weight were negatively associated with MetALD (0.49 [0.26–0.94], *p* = 0.031; 0.50 [0.27–0.93], *p* = 0.029), whereas ASM/BMI was inversely associated with ALD (0.40 [0.18–0.88], *p* = 0.023). The effects of ASM/height^2^, ASM/weight and ASM/BMI on incident MASLD were partially mediated by adiponectin (percentage mediated [95% CI]: 6.3% [2.5%–11.1%]; 9.4% [5.0%–14.6%]; 9.5% [5.1%–15.5%]), uric acid (4.7% [1.6%–8.9%]; 5.3% [2.6%–8.5%]; 5.3% [2.4%–8.8%]), triglyceride (7.1% [3.9%–11.1%]; 7.5% [4.4%–10.9%]; 8.7% [5.3%–13.4%]) and homeostasis model assessment of insulin resistance (13.9% [9.5%–20.4%]; 15.0% [10.0%–20.2%]; 14.5% [9.9%–20.7%]). The effects of ASM/weight and ASM/BMI on incident MASLD were mediated by cholinesterase (8.2% [3.6%–13.1%]; 10.5% [6.1%–16.3%]), prealbumin (6.2% [2.9%–9.8%]; 6.0% [3.0%–10.1%]), retinol‐binding protein‐4 (5.4% [3.0%–8.5%]; 4.6% [1.9%–8.5%]) and osteocalcin (2.1% [0.1%–4.5%]; 2.9% [0.6%–5.7%]).

**Conclusions:**

Relative skeletal muscle mass adjusted for weight or BMI, rather than height alone, better reflects protective effects against SLD. Mediation analysis reveals key metabolic factors linking muscle mass and liver health, offering insights into the pathogenic pathways involved in muscle–liver crosstalk.

## 1 Introduction

Non‐alcoholic fatty liver disease (NAFLD) is the most prevalent metabolic liver disorder globally, affecting approximately one‐third of the general population [1]. Given its inherent limitations regarding exclusionary criteria and stigmatizing terminology, the Delphi consensus recently proposed a new nomenclature–steatotic liver disease (SLD) [2]. SLD encompasses several subtypes, primarily metabolic dysfunction–associated steatotic liver disease (MASLD) and a new subtype, termed metabolic dysfunction and alcohol‐associated liver disease (MetALD) [2]. Emerging evidence indicates these SLD subtypes have distinct prognoses [3, 4, 5], highlighting the need to elucidate etiologic factors to develop prevention and intervention strategies.

Skeletal muscle plays a crucial role in metabolic regulation through both endocrine signalling and substrate metabolism [6, 7]. Low muscle mass is associated with increased risks of cardiovascular disease [8] and mortality [9], but its relationship with novel SLD subtypes remains unclear. In a clinical setting, appendicular skeletal muscle mass (ASM) is widely used to assess skeletal muscle mass and is commonly adjusted for height squared (ASM/height^2^) [10], weight (ASM/weight) [11] or body mass index (ASM/BMI) [12] to reflect relative skeletal muscle mass. We hypothesize that these indices may show differential associations with incident SLD subtypes and progression of liver fibrosis.

Beyond establishing these associations, identifying mediators (e.g., adipokines and metabolic markers) linking muscle mass to SLD may reveal shared biological pathways amenable to intervention. Circulating factors derived from muscle or adipose tissue may serve dual roles as diagnostic biomarkers and therapeutic targets, potentially addressing the unmet clinical need for approved therapies for both muscle atrophy and SLD.

Therefore, using data from a prospective cohort study, we aimed to (1) evaluate the associations of various indices of relative skeletal muscle mass with incident SLD (including its subtypes) and liver fibrosis and (2) identify potential mediators involved in these relationships. This dual approach could help pinpoint high‐risk populations while simultaneously uncovering shared pathophysiological mechanisms that may be amenable to intervention.

## 2 Materials and Methods

### 2.1 Subjects and Study Design

**FIGURE 1:**
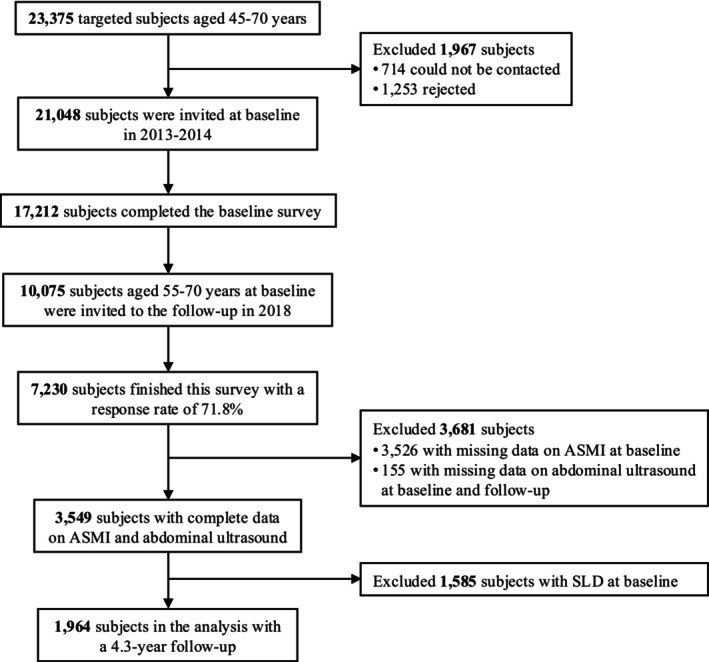
Flowchart of study population. ASMI, appendicular skeletal muscle mass index; SLD, steatosis liver disease.

We analysed data from the Shanghai Nicheng Cohort Study, a population‐based prospective cohort designed to investigate the prevalence, incidence and related factors of cardiometabolic diseases^S1,S2^. The baseline survey was conducted between 2013 and 2014 in Nicheng, a suburban region of Shanghai, China. Among 23 375 eligible residents, 21 408 were invited and 17 212 participants aged 45–70 years completed the baseline assessment. In 2018, 10 075 baseline participants aged 55–70 years were invited for follow‐up, and 7230 attended (response rate: 71.8%). We excluded 3681 individuals who lacked data on appendicular skeletal muscle mass index (ASMI) (*n* = 3526) at baseline or abdominal ultrasound (*n* = 155) at baseline and follow‐up. We further excluded participants with SLD at baseline (*n* = 1585). Finally, 1964 subjects were enrolled with a mean follow‐up duration of 4.3 (standard deviation [SD] 0.36) years (Figure 1).

This study was approved by the ethics committee of the Shanghai Sixth People's Hospital (Approval No: 2015‐27), and written informed consent was obtained from each subject before the study began.

### 2.2 Definition of SLD Subtypes and Liver Fibrosis

Abdominal ultrasound (Z.One Ultra, Zonare Medical Systems Inc., Mountain View, CA, USA) was conducted by experienced ultrasonographers who were unaware of the subjects' conditions. Fatty liver was diagnosed based on known standard criteria, including (1) diffusely increased echogenicity (‘bright’) liver with liver echogenicity greater than kidney or spleen, (2) deep attenuation of ultrasound signal and (3) vascular blurring^S3^.

According to the latest multisociety Delphi consensus statement [2], SLD was defined based on ultrasound evidence of fatty liver. MASLD was defined based on ultrasound evidence of fatty liver, no other causes of hepatic steatosis and in addition to any of the following cardiometabolic risk factors (CMRF): (1) BMI ≥ 23.0 kg/m^2^ (for Asians) or waist circumference (WC) > 90 cm for Asian men and > 80 cm for Asian women; (2) fasting plasma glucose (FPG) ≥ 5.6 mmol/L, or glycated haemoglobin (HbA1c) ≥ 5.7%, or having a history of diabetes; (3) blood pressure ≥ 130/85 mmHg or having a history of hypertension; (4) triglyceride (TG) ≥ 1.70 mmol/L or having lipids lowering treatment; and (5) high‐density lipoprotein cholesterol (HDL‐C) ≤ 1.0 mmol/L for men and ≤ 1.3 mmol/L for women or having lipids lowering treatment [2]. MetALD and alcohol‐associated liver disease (ALD) were both defined based on ultrasound evidence of fatty liver in addition to any of the CMRF. Additionally, MetALD required an alcohol intake of 210–< 420 g/week for men and 140–< 350 g/week for women, whereas ALD required an alcohol intake of ≥ 420 g/week for men and ≥ 350 g/week for women [2].

The fibrosis‐4 (FIB‐4) index^S4^ and NAFLD fibrosis score (NFS)^S5^ were used as surrogate indicators to assess liver fibrosis. Low, intermediate and high fibrosis risk groups were classified based on FIB‐4 and NFS, respectively. The detailed calculation formulas of the two indicators and the cut‐off points for low, intermediate and high fibrosis risk groups are described in Table S1.

### 2.3 Measurement of Body Composition

Skeletal muscle mass was assessed using a Tanita body composition analyser (TBF‐418, Tanita Corp., Tokyo, Japan). ASM was calculated as the total lean mass in both arms and legs. ASMI was evaluated using the following three indices: (1) ASM/height^2^ = ASM (kg)/height^2^ (m); (2) ASM/weight = ASM (kg)/weight (kg) **×** 100%; and (3) ASM/BMI = ASM (kg)/BMI (kg/m^2^). ASM/WC was calculated by dividing ASM (kg) by WC (cm). Appendicular fat mass (AFM) was calculated as the sum of fat mass in both arms and legs, and the muscle/fat ratio (MFR) was created by dividing ASM (kg) by AFM (kg). Magnetic resonance imaging (GE Healthcare, Milwaukee, WI, USA) was used to measure the visceral fat area (VFA) and abdominal subcutaneous fat area (ASFA) from the umbilical slice, which were evaluated using sliceOmatic image analysis software and were detailed in our previous paper^S1^.

### 2.4 Covariates Collection

Data on sex, age, education attainments, smoking, alcohol intake, leisure‐time exercise and medical history (e.g., hypertension and diabetes) were collected via a standardized questionnaire. Height and weight were measured using an established standard protocol^S6^, and BMI was calculated as weight (kg) divided by the square of height (m). WC was measured at the mid‐point between the lower margin of the costal arch and the upper margin of the iliac crest on the mid‐axillary line. Blood pressure was measured twice using a mercury sphygmomanometer, and the average was calculated. Hypertension was defined as average blood pressure ≥ 130/85 mmHg or having a history of hypertension^S7^.

Overnight fasting (at least 10 h) venous blood samples were drawn to measure FPG, HbA1c, fasting insulin (FINS), TG, total cholesterol, HDL‐C, low‐density lipoprotein cholesterol (LDL‐C), alanine aminotransferase, aspartate aminotransferase, gamma‐glutamyl transpeptidase (GGT), cholinesterase (ChE), prealbumin (PA), uric acid (UA), adiponectin, fibroblast growth factor 21 (FGF21), retinol‐binding protein‐4 (RBP4), osteocalcin, hepatitis B surface antigen and hepatitis C virus antibody. The laboratory measurement methods were described in Table S2. Diabetes was defined as FPG ≥ 7.0 mmol/L, or HbA1c ≥ 6.5%, or having a history of diabetes^S8^. Insulin resistance (IR) was determined by the homeostasis model assessment of insulin resistance (HOMA‐IR), as HOMA‐IR = FPG (mmol/L) × FINS (μU/mL)/22.5^S9^.

### 2.5 Statistical Analysis

Descriptive data were presented as medians (25th–75th percentiles) or numbers (proportions) as appropriate. The differences between the two groups were compared using the Kruskal–Wallis rank‐sum test for continuous variables and the chi‐squared test for categorical variables. The *p*‐values were adjusted for multiple hypothesis testing using the Benjamini and Hochberg method. The *p* for trend across ASM/height^2^, ASM/weight and ASM/BMI tertiles was calculated using the Mantel–Haenszel trend test. Correlations of ASM/height^2^, ASM/weight and ASM/BMI with other factors at baseline were evaluated using the partial correlation coefficients adjusted for sex and age.

Modified Poisson regression model with robust error variance or multinomial logistic regression, as appropriate, was used to estimate risk ratios (RRs) and 95% confidence intervals (CIs) for incident SLD, its subtypes and liver fibrosis, as well as for subgroup analysis. The ASM/height^2^, ASM/weight and ASM/BMI were standardized (*z*‐standardization) before entering regression models. By adding the interaction terms to the regression models, potential interactions between ASM/height^2^, ASM/weight, ASM/BMI and the other analysis variables on incident MASLD were tested using the Wald test. In Model 1, sex, age, education attainments (primary school and below or middle school and above), smoking status (never, past or current smokers), drinking status (never, past or current drinkers) and leisure‐time exercise (never, 1–< 30 min/day or ≥ 30 min/day). In Model 2, when assessing the effects of ASM/height^2^ and ASM/weight on outcomes, BMI was further adjusted, whereas WC was additionally adjusted when assessing the effects between ASM/BMI and outcomes, as BMI was a component for calculating ASM/BMI. In Model 3, hypertension (yes or no), diabetes (yes or no), TG and HDL‐C were additionally adjusted. The associations of ASM/height^2^, ASM/weight and ASM/BMI with incident MASLD were also depicted using restricted cubic splines with four knots at the 5th, 35th, 65th and 95th percentiles. The performance of ASM/height^2^, ASM/weight and ASM/BMI in predicting the incidence of MASLD was evaluated using the area under the curve (AUC), net reclassification index (NRI) and integrated discrimination improvement (IDI). For mediation analysis, the average causal mediation effect (ACME) and average direct effect (ADE), which reflected the indirect and direct effects of ASM/height^2^, ASM/weight and ASM/BMI on incident MASLD, were estimated using the ‘mediation’ R package^S10^. These mediation effects were estimated using non‐parametric bootstrapping (1000 simulations). The mediated portion was calculated by dividing ACME by the total effect, which was calculated as ACME plus ADE.

A two‐tailed *p*‐value < 0.05 was considered to be statistically significant. We performed all analyses using SPSS version 26.0 (SPSS Inc., Chicago, Illinois, USA), R version 4.0.2 (R Foundation for Statistical Computing, Vienna, Austria), or StataMP version 14.0 (StataCorp LP, Texas, USA).

## 3 Results

### 3.1 Characteristics of the Study Population

As shown in Table 1, among 1964 participants without SLD at baseline, 598 (30.4%) developed new‐onset SLD during a mean follow‐up of 4.3 years, including 539 MASLD (27.4%), 38 MetALD (1.9%) and 21 ALD (1.1%). Compared with those who did not develop SLD, those with incident MASLD were more likely to be female and had unfavourable metabolic profiles, higher adiposity indicators, FGF21 and RBP4 levels, but lower ASM/height^2^ (medians [25th–75th percentiles]: 7.07 [6.57–8.47] vs. 7.11 [6.41–8.18], *p* = 0.022), ASM/weight (28.7 [26.3–33.9] vs. 31.7 [28.3–35.9], *p* < 0.001), ASM/BMI (0.71 [0.64–0.91] vs. 0.80 [0.69–0.99], *p* < 0.001) and adiponectin. Meanwhile, all individuals with MetALD and ALD were males, having higher GGT, UA, PA, adiposity indicators (BMI, WC and VFA), RBP4 levels and higher ASM/height^2^, ASM/weight and ASM/BMI, but lower adiponectin and osteocalcin than those without SLD.

**TABLE 1:** Baseline characteristics of subjects without and with incident SLD and its subtypes after a 4.3‐year follow‐up.

Characteristic	Total	No SLD^a^	SLD^b^	SLD subtypes			*p*_(a) vs. (b)_	*p*_(a) vs. (c)_	*p*_(a) vs. (d)_	*p*_(a) vs. (e)_
				MASLD^c^	MetALD^d^	ALD^e^				
Numbers (%)	1964	1366 (69.6)	598 (30.4)	539 (27.4)	38 (1.9)	21 (1.1)	—	—	—	—
Soci‐demographic										
Age, years	61.4 (58.4–65.1)	61.3 (58.3–65.0)	61.5 (58.7–65.3)	61.6 (58.7–65.5)	61.2 (58.9–64.8)	60.8 (58.0–65.0)	0.127	0.627	0.951	0.951
Women, no. (%)	1071 (54.5)	729 (53.4)	342 (57.2)	342 (63.5)	0 (0.0)	0 (0.0)	0.129	< 0.001	< 0.001	< 0.001
Middle school or above, *n* (%)	717 (36.5)	510 (37.3)	207 (34.6)	182 (33.8)	16 (42.1)	9 (42.9)	0.271	0.925	0.925	0.925
Lifestyles										
Current smoker, *n* (%)	429 (21.8)	312 (22.8)	117 (19.6)	88 (16.3)	19 (50.0)	10 (47.6)	0.119	0.003	0.001	0.018
Current drinker, *n* (%)	299 (15.2)	220 (16.1)	79 (13.2)	41 (7.6)	23 (60.5)	15 (71.4)	0.115	< 0.001	< 0.001	< 0.001
Physical activity, *n* (%)	93 (4.7)	71 (5.2)	22 (3.7)	18 (3.3)	1 (2.6)	3 (14.3)	0.179	0.186	0.862	0.186
Clinical										
SBP, mmHg	132.0 (122.0–142.5)	131.0 (121.0–142.0)	134.0 (125.0–145.0)	133.5 (125.0–145.0)	137.0 (124.5–146.0)	132.5 (128.0–141.0)	< 0.001	0.001	0.194	0.650
DBP, mmHg	81.0 (77.0–86.0)	81.0 (75.0–85.9)	82.0 (78.6–87.4)	82.0 (78.5–87.0)	81.0 (79.6–89.8)	82.5 (79.0–89.0)	< 0.001	< 0.001	0.348	0.348
FPG, mmol/L	5.7 (5.3–6.2)	5.7 (5.3–6.2)	5.8 (5.4–6.3)	5.8 (5.4–6.2)	6.0 (5.5–6.5)	5.7 (5.4–6.2)	0.005	0.067	0.067	0.805
HbA1c, %	5.7 (5.4–5.9)	5.6 (5.4–5.9)	5.7 (5.5–6.0)	5.7 (5.5–6.0)	5.6 (5.3–6.0)	5.7 (5.4–5.8)	< 0.001	< 0.001	0.878	0.878
FINS, μU/mL	5.6 (4.2–7.7)	5.2 (3.9–6.9)	6.9 (5.0–9.3)	7.0 (5.2–9.5)	6.1 (4.4–7.5)	4.9 (4.3–6.2)	< 0.001	< 0.001	0.205	0.508
HOMA‐IR	1.5 (1.0–2.1)	1.3 (1.0–1.9)	1.8 (1.3–2.4)	1.8 (1.3–2.5)	1.7 (1.1–1.9)	1.3 (1.1–1.6)	< 0.001	< 0.001	0.101	0.567
TG, mmol/L	1.1 (0.8–1.6)	1.0 (0.8–1.4)	1.3 (0.9–1.7)	1.3 (0.9–1.8)	1.3 (0.8–1.7)	1.1 (0.9–1.7)	< 0.001	< 0.001	0.122	0.506
TC, mmol/L	5.0 (4.4–5.6)	5.0 (4.4–5.6)	5.1 (4.5–5.7)	5.1 (4.5–5.7)	5.0 (4.4–5.5)	4.8 (4.2–5.6)	0.025	0.066	0.810	0.810
HDL‐C, mmol/L	1.3 (1.1–1.6)	1.4 (1.1–1.6)	1.3 (1.1–1.5)	1.3 (1.1–1.5)	1.2 (1.1–1.5)	1.3 (1.2–1.5)	< 0.001	< 0.001	0.315	0.956
LDL‐C, mmol/L	3.0 (2.5–3.5)	2.9 (2.5–3.4)	3.1 (2.6–3.6)	3.1 (2.6–3.6)	3.0 (2.5–3.2)	2.8 (2.5–3.6)	< 0.001	< 0.001	0.967	0.967
ALT, U/L	15.0 (12.0–19.0)	14.0 (12.0–19.0)	15.0 (12.0–20.0)	15.0 (12.0–20.0)	15.5 (12.0–19.8)	17.0 (12.0–21.0)	0.025	0.356	0.451	0.451
AST, U/L	22.0 (19.0–25.0)	22.0 (19.0–25.0)	21.0 (19.0–25.0)	21.0 (19.0–24.0)	21.0 (19.0–26.0)	23.0 (20.0–28.0)	0.002	0.004	0.673	0.251
GGT, U/L	20.0 (15.0–29.0)	20.0 (15.0–28.0)	22.0 (17.0–31.0)	21.0 (16.0–29.0)	27.5 (20.0–43.5)	34.0 (28.0–57.0)	< 0.001	0.002	< 0.001	< 0.001
UA, μmol/L	283.0 (238.0–338.0)	276.0 (233.0–329.0)	363.0 (320.0–408.0)	291.0 (245.0–341.0)	354.0 (326.8–382.8)	351.0 (337.0–427.0)	< 0.001	0.002	< 0.001	< 0.001
ChE, U/L	348.0 (305.0–393.0)	338.5 (299.0–385.0)	276.5 (248.0–309.0)	365.5 (321.2–409.0)	358.5 (326.8–403.2)	336.0 (298.0–396.0)	< 0.001	< 0.001	0.125	0.919
PA, mg/L	267.0 (237.0–303.0)	263.0 (234.0–299.0)	296.5 (248.2–349.0)	272.0 (242.0–304.0)	323.5 (288.2–353.2)	318.0 (291.0–351.0)	< 0.001	0.001	< 0.001	< 0.001
Anthropometric										
ASM/height^2^, kg/m^2^	7.15 (6.48–8.32)	7.11 (6.41–8.18)	7.24 (6.62–8.62)	7.07 (6.57–8.47)	8.67 (8.06–9.23)	8.44 (8.12–8.74)	< 0.001	0.022	< 0.001	< 0.001
ASM/weight, %	31.1 (27.8–35.4)	31.7 (28.3–35.9)	29.1 (26.5–34.4)	28.7 (26.3–33.9)	34.5 (33.4–35.6)	34.6 (33.3–35.8)	< 0.001	< 0.001	0.001	0.011
ASM/BMI, m^2^	0.79 (0.67–0.98)	0.80 (0.69–0.99)	0.73 (0.65–0.94)	0.71 (0.64–0.91)	0.96 (0.92–1.02)	0.95 (0.89–1.00)	< 0.001	< 0.001	< 0.001	0.006
BMI, kg/m^2^	23.6 (21.9–25.3)	22.9 (21.2–24.5)	25.0 (23.6–26.8)	25.1 (23.6–26.7)	25.0 (23.4–27.1)	24.0 (23.3–26.2)	< 0.001	< 0.001	< 0.001	0.004
WC, cm	82.0 (76.0–87.0)	80.0 (75.0–86.0)	86.0 (80.0–90.0)	86.0 (80.0–90.0)	87.0 (82.0–94.8)	87.0 (84.0–92.0)	< 0.001	< 0.001	< 0.001	< 0.001
VFA, cm^2^	95.5 (70.3–121.0)	84.6 (63.0–110.8)	114.4 (94.2–142.2)	114.4 (94.5–141.3)	108.5 (79.8–161.2)	121.0 (111.7–133.2)	< 0.001	< 0.001	< 0.001	0.001
ASFA, cm^2^	127.2 (94.1–166.2)	117.7 (86.2–152.5)	150.3 (120.1–188.3)	155.1 (123.4–193.8)	116.5 (92.4–140.9)	109.3 (91.5–146.4)	< 0.001	< 0.001	0.959	0.959
Adipokine										
Adiponectin, μg/mL	4.4 (3.4–5.8)	4.5 (3.5–6.1)	4.1 (3.1–5.3)	4.2 (3.2–5.4)	3.2 (2.3–4.0)	3.9 (2.9–4.6)	< 0.001	< 0.001	< 0.001	0.024
FGF21, pg/mL	213.8 (130.7–334.9)	199.9 (121.1–312.7)	243.7 (154.0–358.8)	239.5 (153.4–352.7)	253.4 (160.1–401.6)	364.5 (188.9–611.5)	< 0.001	< 0.001	0.089	0.008
RBP4, mg/L	45.0 (37.0–55.0)	44.0 (36.0–54.0)	48.0 (41.0–58.0)	47.0 (40.0–56.0)	58.0 (49.2–67.5)	57.0 (47.0–67.0)	< 0.001	< 0.001	< 0.001	< 0.001
Osteocalcin, ng/mL	22.1 (17.3–28.3)	22.5 (17.5–28.6)	21.4 (16.8–28.0)	22.1 (17.3–28.7)	18.3 (15.8–20.4)	15.3 (13.8–18.0)	0.035	0.556	< 0.001	< 0.001

*Note:* Data were presented as medians (25th–75th percentiles) or numbers (proportions) as appropriate.

Abbreviations: ALD, alcohol‐associated liver disease; ALT, alanine aminotransferase; ASFA, abdominal subcutaneous fat area; ASM, appendicular skeletal muscle mass; AST, aspartate aminotransferase; BMI, body mass index; ChE, cholinesterase; DBP, diastolic blood pressure; FGF21, fibroblast growth factor 21; FINS, fasting insulin; FPG, fasting plasma glucose; GGT, gamma‐glutamyl transpeptidase; HbA1c, glycated haemoglobin; HDL‐C, high‐density lipoprotein cholesterol; HOMA‐IR, homeostasis model assessment of insulin resistance; LDL‐C, low‐density lipoprotein cholesterol; MASLD, metabolic‐dysfunction associated steatosis liver disease; MetALD, metabolic dysfunction and alcohol‐associated liver disease; PA, prealbumin; RBP4, retinol‐binding protein‐4; SBP, systolic blood pressure; SLD, steatotic liver disease; TC, total cholesterol; TG, triglyceride; UA, uric acid; VFA, visceral fat area; WC, waist circumference.

As shown in Figure S1, the incidence rates of MASLD and MetALD increased significantly across the ASM/height^2^ tertile in males. Conversely, a significant decrease in the incidence rates of MASLD, MetALD and ALD was observed across the ASM/weight and ASM/BMI tertiles in males. A similar trend in the incidence rate of MASLD across the ASM/height^2^, ASM/weight and ASM/BMI tertiles was observed in females and males. Moreover, there was an increase in most metabolic profiles, adiposity indicators and FGF21 levels, along with a decrease in adiponectin levels across the ASM/height^2^ tertile. However, this trend was reversed in the context of the ASM/weight and ASM/BMI tertiles (Table S3–S5).

### 3.2 Correlations of ASM/Height^2^, ASM/Weight or ASM/BMI With Other Baseline Factors

As shown in Table 2, after adjustment for sex and age, ASM/height^2^ was positively correlated with worse metabolic profiles and adiposity indicators (*r* = 0.05–0.70, all *p* < 0.05) and was negatively correlated with HDL‐C and adiponectin (*r* = −0.09 to −0.22, both *p* < 0.001). In contrast, ASM/weight and ASM/BMI were negatively correlated with worse metabolic profiles, adiposity indicators and RBP4 (*r* = −0.05 to −0.53, all *p* < 0.05) and were positively correlated with HDL‐C, adiponectin and osteocalcin (*r* = 0.08–0.19, all *p* < 0.05). Additionally, there was a weak correlation observed between ASM/height^2^ and both ASM/weight and ASM/BMI (*r* = 0.22 and 0.12, respectively, both *p* < 0.001). However, a strong correlation was evident between ASM/weight and ASM/BMI (*r* = 0.76, *p* < 0.001).

**TABLE 2:** Correlations of ASM/height^2^, ASM/weight and ASM/BMI with other factors at baseline.

Characteristic	ASM/height^2^		ASM/weight		ASM/BMI	
	*r*	*p*	*r*	*p*	*r*	*p*
Clinical						
SBP, mmHg	0.118	< 0.001	−0.130	< 0.001	−0.088	< 0.001
DBP, mmHg	0.114	< 0.001	−0.159	< 0.001	−0.072	0.001
FPG, mmol/L	0.014	0.542	−0.070	0.002	−0.045	0.048
HbA1c, %	0.088	< 0.001	−0.063	0.005	−0.057	0.012
FINS, μU/mL^a^	0.223	< 0.001	−0.328	< 0.001	−0.207	< 0.001
HOMA‐IR^a^	0.205	< 0.001	−0.320	< 0.001	−0.201	< 0.001
TG, mmol/L^a^	0.117	< 0.001	−0.196	< 0.001	−0.163	< 0.001
TC, mmol/L	−0.044	0.050	−0.173	< 0.001	−0.159	< 0.001
HDL‐C, mmol/L	−0.218	< 0.001	0.110	< 0.001	0.075	0.001
LDL‐C, mmol/L	0.054	0.016	−0.190	< 0.001	−0.165	< 0.001
ALT, U/L	0.095	< 0.001	−0.092	< 0.001	−0.071	0.002
AST, U/L	0.005	0.830	0.006	0.780	−0.019	0.400
GGT, U/L	−0.012	0.597	−0.050	0.028	−0.056	0.012
UA, μmol/L^a^	0.087	< 0.001	−0.148	< 0.001	−0.097	< 0.001
ChE, U/L^a^	0.018	0.429	−0.278	< 0.001	−0.210	< 0.001
PA, mg/L^a^	−0.011	0.622	−0.199	< 0.001	−0.129	< 0.001
Anthropometric						
ASM/height^2^, kg/m^2^	1.000	< 0.001	0.216	< 0.001	0.120	< 0.001
ASM/weight, %	0.216	< 0.001	1.000	< 0.001	0.757	< 0.001
ASM/BMI, m^2^	0.120	< 0.001	0.757	< 0.001	1.000	< 0.001
BMI, kg/m^2^	0.699	< 0.001	−0.531	< 0.001	−0.435	< 0.001
WC, cm	0.380	< 0.001	−0.480	< 0.001	−0.239	< 0.001
VFA, cm^2^	0.359	< 0.001	−0.422	< 0.001	−0.231	< 0.001
ASFA, cm^2^	0.332	< 0.001	−0.501	< 0.001	−0.305	< 0.001
Adipokine						
Adiponectin, μg/mL	−0.087	< 0.001	0.188	< 0.001	0.136	< 0.001
FGF21, pg/mL^a^	0.018	0.464	−0.034	0.152	−0.040	0.097
RBP4, mg/L^a^	0.000	0.989	−0.133	< 0.001	−0.081	< 0.001
Osteocalcin, ng/mL	0.007	0.749	0.110	< 0.001	0.080	< 0.001

*Note:* Correlation coefficient was calculated using the partial correlation coefficients adjusted for sex and age.

Abbreviations: ALT, alanine aminotransferase; ASFA, abdominal subcutaneous fat area; ASM, appendicular skeletal muscle mass; AST, aspartate aminotransferase; BMI, body mass index; ChE, cholinesterase; DBP, diastolic blood pressure; FGF21, fibroblast growth factor 21; FINS, fasting insulin; FPG, fasting plasma glucose; GGT, gamma‐glutamyl transpeptidase; HbA1c, glycated haemoglobin; HDL‐C, high‐density lipoprotein cholesterol; HOMA‐IR, homeostasis model assessment of insulin resistance; LDL‐C, low‐density lipoprotein cholesterol; PA, prealbumin; RBP4, retinol‐binding protein‐4; SBP, systolic blood pressure; TC, total cholesterol; TG, triglyceride; UA, uric acid; VFA, visceral fat area; WC, waist circumference.

^a^ Log_e_‐transformed before analysis.

### 3.3 Associations of ASM/Height^2^, ASM/Weight or ASM/BMI With Incident SLD and Its Subtypes

As shown in Table 3, ASM/height^2^ was positively associated with incident SLD in Model 1 (RR per SD 1.67, 95% CI 1.51–1.84) after adjusting for sex, age, education attainments, smoking status, drinking status and leisure‐time exercise. However, this association was reversed after further adjustment for BMI in Model 2 (RR per SD 0.77, 95% CI 0.67–0.89) and remained significant after additional adjustment for hypertension, diabetes, TG and HDL‐C in Model 3 (RR per SD 0.76, 95% CI 0.66–0.88).

**TABLE 3:** Associations of ASM/height^2^, ASM/weight and ASM/BMI (per SD) with incident SLD and its subtypes after a 4.3‐year follow‐up.

Variable	No SLD (*n* = 1366)	SLD (*n* = 598)		SLD subtypes							
				MASLD (*n* = 539)		MetALD (*n* = 38)				ALD (*n* = 21)	
		RR, 95% CI	*p*	RR, 95% CI	*p*	RR, 95% CI		*p*		RR, 95% CI	*p*
ASM/height^2^											
Model 1^a^	Ref.	1.67 (1.51–1.84)	< 0.001	2.30 (1.92–2.76)	< 0.001	1.74 (1.12–2.71)		0.014		1.56 (0.85–2.86)	0.153
Model 2^b^	Ref.	0.77 (0.67–0.89)	< 0.001	0.61 (0.47–0.79)	< 0.001	0.47 (0.25–0.90)		0.022		0.55 (0.23–1.34)	0.189
Model 3^c^	Ref.	0.76 (0.66–0.88)	< 0.001	0.60 (0.47–0.78)	< 0.001	0.49 (0.26–0.94)		0.031		0.54 (0.22–1.32)	0.173
ASM/weight											
Model 1^a^	Ref.	0.47 (0.41–0.54)	< 0.001	0.27 (0.22–0.34)	< 0.001	0.30 (0.16–0.55)		< 0.001		0.39 (0.17–0.89)	0.026
Model 2^b^	Ref.	0.71 (0.61–0.82)	< 0.001	0.59 (0.46–0.76)	< 0.001	0.48 (0.26–0.89)		0.019		0.57 (0.25–1.32)	0.189
Model 3^c^	Ref.	0.71 (0.61–0.82)	< 0.001	0.59 (0.46–0.76)	< 0.001	0.50 (0.27–0.93)		0.029		0.55 (0.23–1.31)	0.177
ASM/BMI											
Model 1^a^	Ref.	0.54 (0.47–0.62)	< 0.001	0.38 (0.30–0.47)	< 0.001	0.62 (0.35–1.09)		0.097		0.40 (0.19–0.86)	0.018
Model 2^b^	Ref.	0.65 (0.57–0.75)	< 0.001	0.49 (0.39–0.62)	< 0.001	0.69 (0.38–1.25)		0.217		0.42 (0.20–0.91)	0.028
Model 3^c^	Ref.	0.66 (0.58–0.76)	< 0.001	0.51 (0.40–0.64)	< 0.001	0.72 (0.39–1.33)		0.294		0.40 (0.18–0.88)	0.023

*Note:* RR (95% CI) was calculated using the multinomial logistic regression models.

Abbreviations: ALD, alcohol‐associated liver disease; ASM, appendicular skeletal muscle mass; BMI, body mass index; CI, confidence interval; HDL‐C, high‐density lipoprotein cholesterol; MASLD, metabolic dysfunction‐associated steatosis liver disease; MetALD, metabolic dysfunction and alcohol‐associated liver disease; RR, risk ratio; SD, standard deviations; SLD, steatotic liver disease; TG, triglyceride; WC, waist circumference.

^a^ Model 1 was adjusted for sex, age, education attainments, smoking status, drinking status and leisure‐time exercise.

^b^ Model 2 was adjusted for variables in Model 1 and also for BMI (ASM/height^2^ and ASM/weight) or WC (ASM/BMI).

^c^ Model 3 was adjusted for variables in Model 2 and also for hypertension, diabetes, TG and HDL‐C.

For SLD subtypes, the association between ASM/height^2^ and MASLD shifted from positive to negative after adjustment for BMI in Model 2 (RR per SD 2.30, 95% CI 1.92–2.76; RR per SD 0.61, 95% CI 0.47–0.79, respectively) and remained significant after further adjustment for metabolic risks in Model 3 (RR per SD 0.60, 95% CI 0.47–0.78). Unlike ASM/height^2^ and MASLD, both ASM/weight and ASM/BMI exhibited consistent negative associations with incident MASLD. In Model 3, each one SD increase in these indices was associated with a 41% and 49% lower risk (RR 0.59, 95% CI 0.46–0.76; RR 0.51, 95% CI 0.40–0.64, respectively). For MetALD, the association between ASM/height^2^ and incident MetALD changed from positive to negative after adjustment for BMI (RR per SD 1.74, 95% CI 1.12–2.71; RR per SD 0.47, 95% CI 0.25–0.90, respectively). Meanwhile, the risk of MetALD was significantly halved with each one SD increase in ASM/weight (RR 0.50, 95% CI 0.27–0.93, Model 3), but no association was found between ASM/BMI and incident MetALD. For ALD, a negative association was observed between ASM/BMI and incident ALD (RR per SD 0.40, 95% CI 0.18–0.88, Model 3), but no significant associations were identified between both ASM/height^2^ and ASM/weight and incident ALD (RR per SD 0.54, 95% CI 0.22–1.32; RR per SD 0.55, 95% CI 0.23–1.31, respectively; Model 3). Additionally, associations between ASM/WC and incident SLD and its subtypes were similar to those observed for ASM/BMI (Table S6).

In addition, ASM/weight and ASM/BMI exhibited linearly inverse associations with incident MASLD (Figure S2). Conversely, ASM/height^2^ initially showed a linear positive association with incident MASLD (Model 1; Figure S2), but this association was reversed and no longer maintained linearity after adjusting for BMI or metabolic risks (Models 2–3; Figure S2).

### 3.4 Performance of ASM/Height^2^, ASM/Weight or ASM/BMI in Predicting Incident MASLD

As shown in Table S7, in the sex‐ and age‐adjusted models, adding ASM/weight significantly improved the prediction of MASLD incidence compared with ASM/height^2^, ASM/BMI and ASM/WC (AUC: 0.693 vs. 0.653, 0.645 and 0.589; NRI: 55.6% vs. 42.1%, 34.9% and 21.1%; IDI: 7.8% vs. 4.5%, 3.9% and 0.8%, all *p* < 0.001). Additionally, MFR exhibited superior predictive performance over ASM/height^2^, ASM/weight, ASM/BMI and ASM/WC for the occurrence of MASLD (AUC = 0.730, NRI = 72.4%, IDI = 9.2% for MFR).

### 3.5 Mediation by Metabolic Factors and Adipokines

As shown in Table 4, the proportion of the effects of ASM/height^2^, ASM/weight and ASM/BMI on the risk of MASLD mediated by HOMA‐IR was 13.9% (95% CI 9.5%–20.4%), 15.0% (10.0%–20.2%) and 14.5% (9.9%–20.7%); 7.1% (3.9%–11.1%), 7.5% (4.4%–10.9%) and 8.7% (5.3%–13.4%) for TG; 4.7% (1.6%–8.9%), 5.3% (2.6%–8.5%) and 5.3% (2.4%–8.8%) for UA; and 6.3% (2.5%–11.1%), 9.4% (5.0%–14.6%) and 9.5% (5.1%–15.5%) for adiponectin. The percentages of the effects of ASM/weight and ASM/BMI on incident MASLD mediated by ChE were 8.2% (3.6%–13.1%) and 10.5% (6.1%–16.3%); 6.2% (2.9%–9.8%) and 6.0% (3.0%–10.1%) for PA; 5.4% (3.0%–8.5%) and 4.6% (1.9%–8.5%) for RBP4; and 2.1% (0.1%–4.5%) and 2.9% (0.6%–5.7%) for osteocalcin. Additionally, FGF21 mediated 2.8% (0.2%–6.2%) of the effect of ASM/BMI on incident MASLD, whereas only marginal mediation was observed for ASM/height^2^ and ASM/weight (3.4%, −0.1% to 8.7%, *p* = 0.054; 2.1%, −0.0% to 4.4%, *p* = 0.052, respectively). Other factors, including SBP, DBP, FINS, HDL‐C and LDL‐C, also mediated the associations of ASM/height^2^, ASM/weight and ASM/BMI with MASLD (Table S8).

**TABLE 4:** Mediation analyses to estimate the indirect, direct and total effects of ASM/height^2^, ASM/weight and ASM/BMI on incident MASLD.

Mediator	ACME^b^ Estimate (95% CI)	ADE^b^ Estimate (95% CI)	Total effect^b^ Estimate (95% CI)	Percentage mediated % (95% CI)	*p* ^c^
ASM/height^2^					
HOMA‐IR^a^	0.03 (0.02–0.04)	0.19 (0.13–0.26)	0.22 (0.16–0.30)	13.9 (9.5–20.4)	< 0.001
TG, mmol/L^a^	0.02 (0.009–0.02)	0.21 (0.16–0.28)	0.23 (0.17–0.30)	7.1 (3.9–11.1)	< 0.001
UA, μmol/L^a^	0.01 (0.004–0.02)	0.22 (0.16–0.29)	0.23 (0.17–0.30)	4.7 (1.6–8.9)	< 0.001
ChE, U/L^a^	0.003 (−0.004 to 0.01)	0.23 (0.17–0.31)	0.24 (0.17–0.32)	1.4 (−1.9 to 4.5)	0.362
PA, mg/L^a^	−0.001 (−0.008 to 0.006)	0.23 (0.17–0.31)	0.23 (0.17–0.31)	−0.2 (−3.4 to 2.6)	0.896
Adiponectin, μg/mL	0.02 (0.006–0.03)	0.23 (0.16–0.31)	0.25 (0.18–0.33)	6.3 (2.5–11.1)	< 0.001
RBP4, mg/L^a^	0.001 (−0.006 to 0.008)	0.23 (0.17–0.30)	0.23 (0.17–0.30)	0.4 (−2.6 to 3.7)	0.776
Osteocalcin, ng/mL^a^	0.001 (−0.003 to 0.005)	0.23 (0.17–0.30)	0.23 (0.17–0.30)	0.3 (−1.5 to 2.1)	0.758
FGF21, pg/mL^a^	0.008 (−0.000 to 0.02)	0.22 (0.15–0.30)	0.23 (0.16–0.31)	3.4 (−0.1 to 8.7)	0.054
ASM/weight					
HOMA‐IR^a^	−0.02 (−0.03 to −0.02)	−0.14 (−0.17 to −0.11)	−0.16 (−0.19 to −0.14)	15.0 (10.0–20.2)	< 0.001
TG, mmol/L^a^	−0.01 (−0.02 to −0.007)	−0.15 (−0.18 to −0.13)	−0.16 (−0.19 to −0.14)	7.5 (4.4–10.9)	< 0.001
UA, μmol/L^a^	−0.009 (−0.01 to −0.004)	−0.15 (−0.18 to −0.13)	−0.16 (−0.19 to −0.13)	5.3 (2.6–8.5)	< 0.001
ChE, U/L^a^	−0.01 (−0.02 to −0.006)	−0.15 (−0.18 to −0.12)	−0.16 (−0.19 to −0.14)	8.2 (3.6–13.1)	0.002
PA, mg/L^a^	−0.010 (−0.02 to −0.005)	−0.15 (−0.19 to −0.13)	−0.16 (−0.20 to −0.14)	6.2 (2.9–9.8)	< 0.001
Adiponectin, μg/mL	−0.02 (−0.02 to −0.008)	−0.15 (−0.19 to −0.12)	−0.16 (−0.20 to −0.13)	9.4 (5.0–14.6)	< 0.001
RBP4, mg/L^a^	−0.009 (−0.01 to −0.005)	−0.15 (−0.19 to −0.13)	−0.16 (−0.20 to −0.14)	5.4 (3.0–8.5)	< 0.001
Osteocalcin, ng/mL^a^	−0.003 (−0.007 to 0.000)	−0.15 (−0.19 to −0.13)	−0.16 (−0.19 to −0.13)	2.1 (0.1–4.5)	0.040
FGF21, pg/mL^a^	−0.003 (−0.007 to 0.000)	−0.16 (−0.19 to −0.13)	−0.16 (−0.19 to −0.13)	2.1 (−0.0 to 4.4)	0.052
ASM/BMI					
HOMA‐IR^a^	−0.02 (−0.03 to −0.01)	−0.12 (−0.15 to −0.09)	−0.14 (−0.17 to −0.11)	14.5 (9.9–20.7)	< 0.001
TG, mmol/L^a^	−0.01 (−0.02 to −0.007)	−0.13 (−0.16 to −0.10)	−0.14 (−0.17 to −0.12)	8.7 (5.3–13.4)	< 0.001
UA, μmol/L^a^	−0.007 (−0.01 to −0.003)	−0.13 (−0.16 to −0.11)	−0.14 (−0.17 to −0.11)	5.3 (2.4–8.8)	< 0.001
ChE, U/L^a^	−0.01 (−0.02 to −0.009)	−0.13 (−0.15 to −0.10)	−0.14 (−0.17 to −0.11)	10.5 (6.1–16.3)	< 0.001
PA, mg/L^a^	−0.009 (−0.01 to −0.004)	−0.13 (−0.16 to −0.11)	−0.14 (−0.17 to −0.11)	6.0 (3.0–10.1)	< 0.001
Adiponectin, μg/mL	−0.01 (−0.02 to −0.007)	−0.13 (−0.17 to −0.11)	−0.15 (−0.18 to −0.12)	9.5 (5.1–15.5)	< 0.001
RBP4, mg/L^a^	−0.006 (−0.01 to −0.002)	−0.13 (−0.16 to −0.11)	−0.14 (−0.17 to −0.11)	4.6 (1.9–8.5)	0.004
Osteocalcin, ng/mL^a^	−0.004 (−0.008 to −0.001)	−0.13 (−0.16 to −0.11)	−0.14 (−0.17 to −0.11)	2.9 (0.6–5.7)	0.012
FGF21, pg/mL^a^	−0.004 (−0.009 to −0.000)	−0.14 (−0.17 to −0.12)	−0.15 (−0.18 to −0.12)	2.8 (0.2–6.2)	0.036

Abbreviations: ACME, average causal mediation effects; ADE, average direct effects; ASM, appendicular skeletal muscle mass; BMI, body mass index; ChE, cholinesterase; CI, confidence interval; FGF21, fibroblast growth factor 21; HOMA‐IR, homeostasis model assessment of insulin resistance; MASLD, metabolic‐dysfunction associated steatosis liver disease; PA, prealbumin; RBP4, retinol‐binding protein‐4; SD, standard deviations; TG, triglyceride; UA, uric acid.

^a^ Log_e_‐transformed before analysis.

^b^ Two multivariable‐adjusted regression models (linear regression model for the mediator and Poisson regression model for the outcome) were established to evaluate these effects. The effects of ASM/height^2^, ASM/weight and ASM/BMI (per SD) on incident MASLD were adjusted for sex, age, education attainments, smoking status, drinking status and leisure‐time exercise. CIs were calculated using percentile bootstrap (replications = 1000).

^c^ *p*‐value for percentage mediated.

### 3.6 Associations of ASM/Height^2^, ASM/Weight or ASM/BMI With Incident MASLD Among Subgroups

Subgroup analyses were conducted according to age, sex, hypertension and diabetes, respectively. As shown in Figure S3, the positive associations of ASM/height^2^ (RR per SD 1.34–1.79) and negative associations of ASM/weight (RR per SD 0.29–0.73) and ASM/BMI (RR per SD 0.40–0.76) with incident MASLD were all observed in those subgroups, irrespective of age, sex and hypertension and in subgroups without diabetes. In addition, a significant interaction was identified between ASM/height^2^, ASM/weight, ASM/BMI and age (*p* for interaction < 0.001; *p* = 0.006; *p* = 0.003, respectively).

We further performed analyses stratified by obesity indices (BMI, WC, VFA and ASFA). As shown in Figure S4, the associations of ASM/height^2^, ASM/weight and ASM/BMI with incident MASLD were evident across all subgroups, except in the obese subgroup as defined by BMI.

### 3.7 Associations of ASM/Height^2^, ASM/Weight or ASM/BMI With Liver Fibrosis

As shown in Figure S5, the proportions of SLD individuals with low, intermediate and high fibrosis risk at follow‐up increased significantly across the ASM/height^2^ tertile and decreased markedly across the ASM/weight and ASM/BMI tertiles. With the non‐SLD as a reference, when assessing the liver fibrosis using FIB‐4, negative associations of ASM/height^2^, ASM/weight and ASM/BMI with different liver fibrosis stages were observed (RR per SD 0.29–0.63), except for the association between ASM/BMI and the high fibrosis risk group (Model 2; Table S9). When using NFS to define liver fibrosis, increasing ASM/height^2^, ASM/weight and ASM/BMI were negatively associated with different liver fibrosis stages (RR per SD 0.29–0.69) (Model 2; Table S10).

## 4 Discussion

### 4.1 Major Findings

To our knowledge, this is the first prospective cohort study to examine the associations between multiple skeletal muscle mass indices—ASM/height^2^, ASM/weight and ASM/BMI—and the newly proposed SLD and its subtypes, while also exploring potential mediators of these associations. Our findings demonstrated that higher ASM/weight and ASM/BMI were associated with around 40% reduced risk of new‐onset SLD and MASLD. Notably, the association of ASM/height^2^ with SLD, MASLD and MetALD shifted from positive to negative after adjustment for BMI. Meanwhile, increased ASM/weight was inversely associated with MetALD risk and higher ASM/BMI was associated with a reduced risk of ALD.

### 4.2 Skeletal Muscle Mass and SLD Subtypes Risks

So far, only four studies—one from the United Kingdom [13] and three from Korea [14, 15, 16]—have prospectively examined ASM/weight [13, 14, 15] or ASM/BMI [16] in relation to incident NAFLD, consistently reporting protective effects. However, the prospective impact of ASM/height^2^ remains unexplored, and no studies have evaluated skeletal muscle mass indices in relation to newly defined SLD subtypes. Our study addresses these gaps, demonstrating that higher ASM/weight and ASM/BMI are inversely associated with MASLD risk.

Notably, higher ASM/height^2^ was initially associated with increased risks of SLD, MASLD and MetALD, but this relationship reversed after BMI adjustment. A similar pattern was observed with ASM, supporting the confounding role of adiposity in these associations. Using MFR also yielded consistent results, with higher MFR associated with reduced MASLD risk (Table S11).

We also observed that the association between ASM/height^2^ and MASLD risk was not observed among obese individuals defined by BMI (Figure S4), suggesting its limitation in stratifying obesity‐related risk. Notably, ASM/height^2^ showed opposing effects in lean (BMI ≤ 23 kg/m^2^) versus non‐lean MASLD: protective in the former and detrimental in the latter (Table S12). This may reflect that absolute skeletal muscle mass tends to increase with body weight, making ASM/height^2^ less informative among individuals with obesity.

In addition, our study was the first to examine relationships between skeletal muscle mass indices and SLD with different etiologies, such as alcohol intake. Higher ASM/height^2^ and ASM/weight were inversely associated with reduced risks of MetALD and higher ASM/BMI was protective against ALD. Although the number of MetALD and ALD cases was limited, these findings warrant further investigation in larger cohorts. Regarding liver fibrosis, existing studies relied on cross‐sectional data [17, 18], with limited prospective evidence [16]. Our findings added to this literature, indicating a potential protective effect of these skeletal muscle mass indices against liver fibrosis (Table S9–S10).

### 4.3 Mediators of Muscle–Liver Crosstalk

Our findings suggested that skeletal muscle health influenced the development and progression of SLD, highlighting the need for therapeutic strategies that target pathophysiological mechanisms shared between muscle and liver. Notably, the effects of skeletal muscle mass on incident MAFLD were predominantly mediated by IR, accounting for 13.9%–15.0% of the total effect. IR is known to promote proteolysis and muscle wasting [19] and also induces lipolysis, resulting in non‐esterified fatty acids (NEFAs) that are taken up by muscle and liver, contributing to lipotoxicity and NAFLD pathogenesis [20].

We also identified several metabolic mediators—UA, ChE and PA—linking skeletal muscle and liver. UA, a product of purine metabolism, plays dual roles as both an oxidant and an antioxidant. Research on the relationship between UA and skeletal muscle mass has yielded inconsistent conclusions [21, 22]. At normal levels, UA might be a protective factor against oxidative stress in skeletal muscle, but elevated concentrations are associated with increased oxidative stress [23], which has been recognized in NAFLD patients in both animal experiments and clinical studies [24]. Our findings indicated an inverse correlation between skeletal muscle mass and UA, with mediation analysis implying that around 5% of the effects of skeletal muscle mass on MASLD were mediated by UA. ChE, also known as butyrylcholinesterase (BChE), is an α‐glycoprotein primarily synthesized in the liver [25]. BChE levels declined in acute and chronic liver damage, cirrhosis, hepatocellular carcinoma and protein–energy malnutrition [25, 26], being a biomarker for liver impairment and malnutrition. Conversely, BChE levels increased in individuals with NAFLD [27]. It is speculated that in patients with NAFLD, the increased availability of NEFAs to the liver may stimulate the production of butyryl‐CoA and butyrylcholine, and that this increase may be associated with elevated BChE activity [28]. Similar to BChE, PA is also a glycoprotein, mainly secreted by the liver. Clinically, decreased PA was associated with malnutrition or liver damage, whereas increased PA has been proven to be correlated with IR and obesity [29, 30]. Our study demonstrated a positive association between increased BChE or PA and MASLD risk (Table S13), mediating 8.2%–10.5% and 6.0%–6.2% of the effects of skeletal muscle mass on MASLD, respectively. As BChE and PA are nutritional biomarkers, their increased levels may reflect over‐nutrition, adversely affecting both muscle and liver health. Routine monitoring of these markers may aid early detection and management of skeletal muscle wasting and MASLD.

Adipokines also mediated the associations between muscle and liver in our study. Adiponectin, an adipokine with many beneficial effects, promotes glucose uptake in skeletal muscle and, in the liver, reduces the influx of NEFAs, enhances fatty acid oxidation and improves insulin sensitivity [31, 32]. Our findings indicated that around 10% of the effect of skeletal muscle mass on MASLD was mediated by increased adiponectin. RBP4, secreted by hepatocytes and adipose tissue [33], promotes muscle atrophy through specific inhibitors targeting the signalling receptor and transporter of retinol 6‐dependent and Janus kinase 2/signal transducer and activator of transcription 3 pathway‐mediated mechanism in denervated skeletal muscle [34]. RBP4 was inversely related to the adipocyte glucose transporter 4, which plays a key role in muscle and liver IR [33, 35], a well‐known pathophysiological hallmark of NAFLD. Osteocalcin, a bone‐derived hormone, promotes protein synthesis, and administering exogenous osteocalcin is sufficient to increase muscle mass in aging mice [36]. Meanwhile, osteocalcin is negatively associated with NAFLD development [37]. Our study demonstrated that increased RBP4 and decreased osteocalcin partially mediated the effects of skeletal muscle mass on MASLD. FGF21, as an endocrine hormone, improves hepatic and peripheral insulin sensitivity [38] and reduces TG and diacylglycerol levels in the liver and skeletal muscle in mice [39]. Despite these metabolic benefits, elevated FGF21 levels in metabolic disorders may reflect FGF21 resistance or a compensatory response to metabolic stress [40]. Our findings indicated that FGF21 mediated 2.8% of the effects of relative skeletal muscle mass (only defined by ASM/BMI) on incident MASLD.

Collectively, these findings provide mechanistic insights into muscle–liver crosstalk and suggest potential therapeutic targets. Although individual mediators explained a modest proportion of the associations (ranging from 2.1% to 15.0%), this is consistent with the complex, multifactorial nature of metabolic diseases, where multiple small effects may accumulate to produce clinically significant outcomes. Future studies should explore additional mediators and investigate potential additive or synergistic effects in this multifactorial context.

### 4.4 Strengths and Limitations

Our study had several notable strengths. First, this was the first community‐based prospective cohort study to simultaneously examine skeletal muscle mass—ASM/height^2^, ASM/weight and ASM/BMI—in relation to new‐onset SLD subtypes. Second, we identified several novel mediators (UA, ChE, PA, adiponectin, RBP4 and osteocalcin), which may be involved in the effects of skeletal muscle mass on MASLD. Third, a broad range of potential confounders was adjusted for.

We admitted that this study had limitations. First, although ultrasound has limited sensitivity in diagnosing fatty liver^S11^, it is still recommended as the preferred imaging modality to detect fatty liver in large‐scale studies^S12,S13^. Second, liver fibrosis was evaluated using FIB‐4 and NFS, which have been validated as acceptable surrogates for assessing fibrosis in epidemiological studies^S14,S15^. Third, skeletal muscle mass was measured using BIA rather than the gold standard dual‐energy X‐ray absorptiometry; however, BIA might be more appropriate for clinical studies given its low cost and accessibility. Fourth, certain confounders, such as dietary factors, were not evaluated. Fifth, since data on virus hepatitis were unavailable at follow‐up, we could not analyse these data. Nevertheless, cross‐sectional analyses indicated protective effects of ASM/height^2^, ASM/weight and ASM/BMI on prevalent other aetiology SLD (Figure S6 and Table S14). Sixth, our findings may be subject to potential selection bias, as the final analysis included a small subset of the original cohort. However, sensitivity analyses supported the robustness of our results (Table S15). Finally, the fact that our study was conducted in middle‐aged and elderly Chinese limited the generalizability of those results.

In conclusion, our findings indicate that skeletal muscle mass relative to weight, rather than height alone, may better reflect its protective effects against SLD risk. Besides, ASM/weight was found to be superior to ASM/height^2^ and ASM/BMI in predicting incident MASLD. Our findings provide valuable evidence for selecting optimal metrics of skeletal muscle mass and support its role in preventing incident SLD subtypes and potentially liver fibrosis. These identified mediators provide some new avenues for future research on muscle–liver crosstalk.

## Conflicts of Interest

The authors declare no conflicts of interest.

## Supporting information


**Figure S1:** Incidence rates of SLD subtypes stratified by ASM/height^2^, ASM/weight and ASM/BMI.
**Figure S2:** Associations of ASM/height2 (A–C), ASM/weight (D–F) and ASM/BMI (G–I) with incident MASLD.
**Figure S3:** Associations of ASM/height2, ASM/weight and ASM/BMI (per SD) with incident MASLD among subgroups.
**Figure S4:** Associations of ASM/height2, ASM/weight and ASM/BMI (per SD) with incident MASLD stratified by obesity indices.
**Figure S5:** Proportions of liver fibrosis at follow‐up assessed by FIB‐4 (A) and NFS (B) stratified by ASM/height2, ASM/weight and ASM/BMI*.
**Figure S6:** Flowchart of study population for cross‐sectional analysis.
**Table S1:** Risk models for liver fibrosis.
**Table S2:** Summary of laboratory tests.
**Table S3:** Baseline characteristics of subjects stratified by ASM/height2*.
**Table S4:** Baseline characteristics of subjects stratified by ASM/weight*.
**Table S5:** Baseline characteristics of subjects stratified by ASM/BMI*.
**Table S6:** Associations of ASM/WC (per SD) with incident SLD and its subtypes after a 4.3‐year follow‐up.
**Table S7:** Comparisons of incremental values among ASM/height2, ASM/weight, ASM/BMI and MFR in predicting the incidence of MASLD.
**Table S8:** Mediation analyses to estimate the indirect, direct and total effects of ASM/height2, ASM/weight and ASM/BMI on incident MASLD.
**Table S9:** Associations of ASM/height2, ASM/weight and ASM/BMI (per SD) with incident liver fibrosis evaluated by FIB‐4 (n = 1660)*.
**Table S10:** Associations of ASM/height2, ASM/weight and ASM/BMI (per SD) with incident liver fibrosis evaluated by NFS (n = 1746)*.
**Table S11:** Associations of ASM and MFR (per SD) with incident SLD and its subtypes.
**Table S12:** Associations of ASM/height2, ASM/weight and ASM/BMI (per SD) with incident lean MASLD* or non‐lean MASLD.
**Table S13:** Associations between baseline characteristics (per SD) and incident MASLD.
**Table S14:** Associations of ASM/height2, ASM/weight and ASM/BMI (per SD) with prevalent SLD and its subtypes based on cross‐sectional data (n = 8427).
**Table S15:** Associations of ASM/height2, ASM/weight and ASM/BMI (per SD) with incident SLD and its subtypes after imputing missing data on body composition.*

## Data Availability

De‐identified data in our study will not be made available publicly. For further detailed data access policy and procedure, please contact the corresponding author.
