# Supplementary material for: Effect of Skeletal Muscle Mass and Its Associated Mediators on the Development of Steatotic Liver Disease: A Cohort Study in China
Source: J Cachexia Sarcopenia Muscle. 2025 Nov 4;16(6):e70093. doi: 10.1002/jcsm.70093 (PMC12583972; doi:10.1002/jcsm.70093)
Supplement: Supplementary file 1 — Figure S1: Incidence rates of SLD subtypes stratified by ASM/height2, ASM/weight and ASM/BMI. Figure S2: Associations of ASM/height2 (A–C), ASM/weight (D–F) and ASM/BMI (G–I) with incident MASLD. Figure S3: Associations of ASM/height2, ASM/weight and ASM/BMI (per SD) with incident MASLD among subgroups. Figure S4: Associations of ASM/height2, ASM/weight and ASM/BMI (per SD) with incident MASLD stratified by obesity indices. Figure S5: Proportions of liver fibrosis at follow‐up assessed by FIB‐4 (A) and NFS (B) stratified by ASM/height2, ASM/weight and ASM/BMI*. Figure S6: Flowchart of study population for cross‐sectional analysis. Table S1: Risk models for liver fibrosis. Table S2: Summary of laboratory tests. Table S3: Baseline characteristics of subjects stratified by ASM/height2*. Table S4: Baseline characteristics of subjects stratified by ASM/weight*. Table S5: Baseline characteristics of subjects stratified by ASM/BMI*. Table S6: Associations of ASM/WC (per SD) with incident SLD and its subtypes after a 4.3‐year follow‐up. Table S7: Comparisons of incremental values among ASM/height2, ASM/weight, ASM/BMI and MFR in predicting the incidence of MASLD. Table S8: Mediation analyses to estimate the indirect, direct and total effects of ASM/height2, ASM/weight and ASM/BMI on incident MASLD. Table S9: Associations of ASM/height2, ASM/weight and ASM/BMI (per SD) with incident liver fibrosis evaluated by FIB‐4 (n = 1660)*. Table S10: Associations of ASM/height2, ASM/weight and ASM/BMI (per SD) with incident liver fibrosis evaluated by NFS (n = 1746)*. Table S11: Associations of ASM and MFR (per SD) with incident SLD and its subtypes. Table S12: Associations of ASM/height2, ASM/weight and ASM/BMI (per SD) with incident lean MASLD* or non‐lean MASLD. Table S13: Associations between baseline characteristics (per SD) and incident MASLD. Table S14: Associations of ASM/height2, ASM/weight and ASM/BMI (per SD) with prevalent SLD and its subtypes based on cross‐sectional data [file JCSM-16-e70093-s001.docx]

**Effect of skeletal muscle mass and its associated mediators on the development of steatotic liver disease: a cohort study in China**

[Figure S1. Incidence rates of SLD subtypes stratified by ASM/height^2^, ASM/weight, and ASM/BMI. 3](#_Toc201256489)

[Figure S2. Associations of ASM/height^2^ (A-C), ASM/weight (D-F), and ASM/BMI (G-I) with incident MASLD. 4](#_Toc201256490)

[Figure S3. Associations of ASM/height^2^, ASM/weight, and ASM/BMI (per SD) with incident MASLD among subgroups. 5](#_Toc201256491)

[Figure S4. Associations of ASM/height^2^, ASM/weight, and ASM/BMI (per SD) with incident MASLD stratified by obesity indices. 6](#_Toc201256492)

[Figure S5. Proportions of liver fibrosis at follow-up assessed by FIB-4 (A) and NFS (B) stratified by ASM/height^2^, ASM/weight, and ASM/BMI^*^. 7](#_Toc201256493)

[Figure S6. Flowchart of study population for cross-sectional analysis. 8](#_Toc201256494)

[Table S1. Risk models for liver fibrosis. 9](#_Toc201256495)

[Table S2. Summary of laboratory tests. 10](#_Toc201256496)

[Table S3. Baseline characteristics of subjects stratified by ASM/height^2*^. 11](#_Toc201256497)

[Table S4. Baseline characteristics of subjects stratified by ASM/weight^*^. 12](#_Toc201256498)

[Table S5. Baseline characteristics of subjects stratified by ASM/BMI^*^. 13](#_Toc201256499)

[Table S6. Associations of ASM/WC (per SD) with incident SLD and its subtypes after a 4.3-year follow-up. 14](#_Toc201256500)

[Table S7. Comparisons of incremental values among ASM/height^2^, ASM/weight, ASM/BMI, and MFR in predicting the incidence of MASLD. 15](#_Toc201256501)

[Table S8. Mediation analyses to estimate the indirect, direct, and total effects of ASM/height^2^, ASM/weight, and ASM/BMI on incident MASLD. 16](#_Toc201256502)

[Table S9. Associations of ASM/height^2^, ASM/weight, and ASM/BMI (per SD) with incident liver fibrosis evaluated by FIB-4 (n = 1660)^*^. 18](#_Toc201256503)

[Table S10. Associations of ASM/height^2^, ASM/weight, and ASM/BMI (per SD) with incident liver fibrosis evaluated by NFS (n = 1746)^*^. 19](#_Toc201256504)

[Table S11. Associations of ASM and MFR (per SD) with incident SLD and its subtypes. 20](#_Toc201256505)

[Table S12. Associations of ASM/height^2^, ASM/weight, and ASM/BMI (per SD) with incident lean MASLD^*^ or non-lean MASLD. 21](#_Toc201256506)

[Table S13. Associations between baseline characteristics (per SD) and incident MASLD. 22](#_Toc201256507)

[Table S14. Associations of ASM/height^2^, ASM/weight, and ASM/BMI (per SD) with prevalent SLD and its subtypes based on cross-sectional data (n = 8427). 23](#_Toc201256508)

[Table S15. Associations of ASM/height^2^, ASM/weight, and ASM/BMI (per SD) with incident SLD and its subtypes after imputing missing data on body composition.^*^ 24](#_Toc201256509)

[Reference 25](#_Toc201256510)


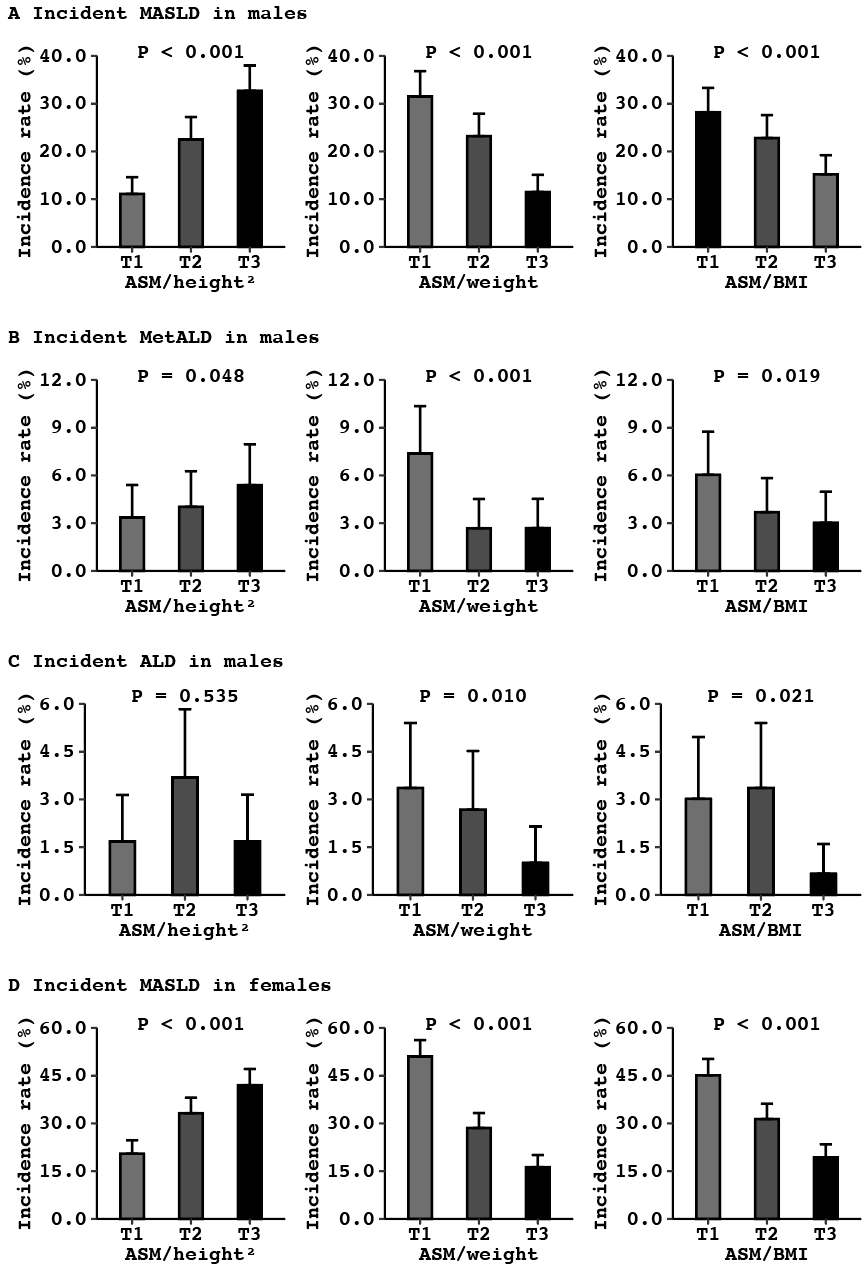


# Figure S1. Incidence rates of SLD subtypes stratified by ASM/height^2^, ASM/weight, and ASM/BMI.

P value for trend.

ALD, alcohol-associated liver disease; ASM, appendicular skeletal muscle mass; BMI, body mass index; MASLD, metabolic-dysfunction associated steatosis liver disease; MetALD, metabolic dysfunction and alcohol-associated liver disease; SLD, steatotic liver disease; T1-T3, tertile 1-tertile 3.


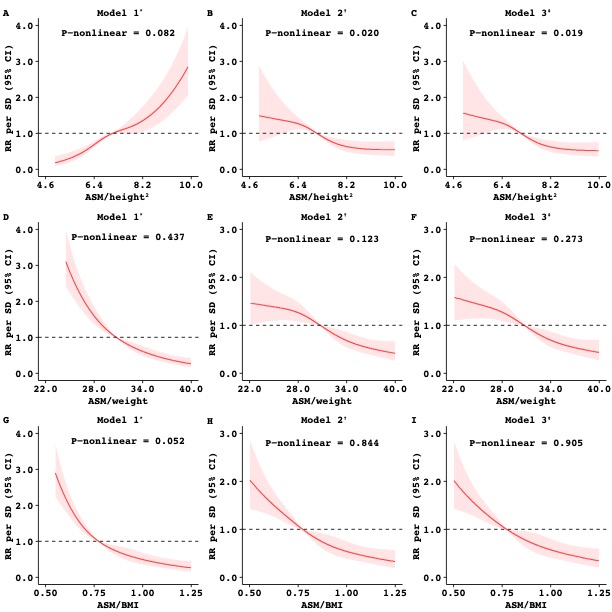


# Figure S2. Associations of ASM/height^2^ (A-C), ASM/weight (D-F), and ASM/BMI (G-I) with incident MASLD.

RR (95% CI) per SD was calculated using the modified Poisson regression model with robust error variance.

^*^Model 1 was adjusted for sex, age, education attainments, smoking status, drinking status, and leisure-time exercise.

^†^Model 2 was adjusted for variables in Model 1 and also for BMI (ASM/height^2^ and ASM/weight) or WC (ASM/BMI).

^‡^Model 3 was adjusted for variables in Model 2 and also for hypertension, diabetes, TG, and HDL-C.

ASM, appendicular skeletal muscle mass; BMI, body mass index; CI, confidence interval; HDL-C, high-density lipoprotein cholesterol; MASLD, metabolic-dysfunction associated steatosis liver disease; RR, risk ratio; SD, standard deviations; TG, triglyceride; WC, waist circumference.


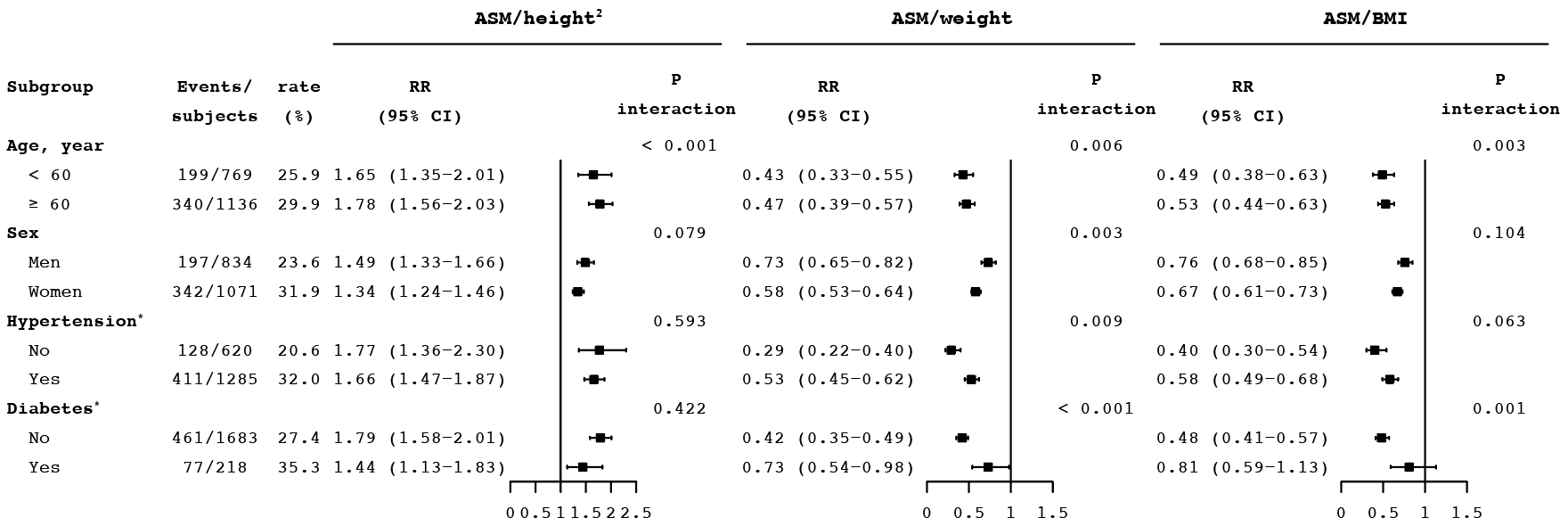


# Figure S3. Associations of ASM/height^2^, ASM/weight, and ASM/BMI (per SD) with incident MASLD among subgroups.

RR (95% CI) was calculated using the modified Poisson regression model with robust error variance adjusting for sex, age, education attainments, smoking status, drinking status, and leisure-time exercise.

^*^Hypertension was defined as blood pressure ≥ 130/85 mmHg or with a history of hypertension; diabetes was defined as FPG ≥ 7.0 mmol/L or HbA1c ≥ 6.5%, or with a history of diabetes.

ASM, appendicular skeletal muscle mass; BMI, body mass index; CI, confidence interval; FPG, fasting plasma glucose; HbA1c, glycated hemoglobin; MASLD, metabolic-dysfunction associated steatosis liver disease; RR, risk ratio; SD, standard deviations.


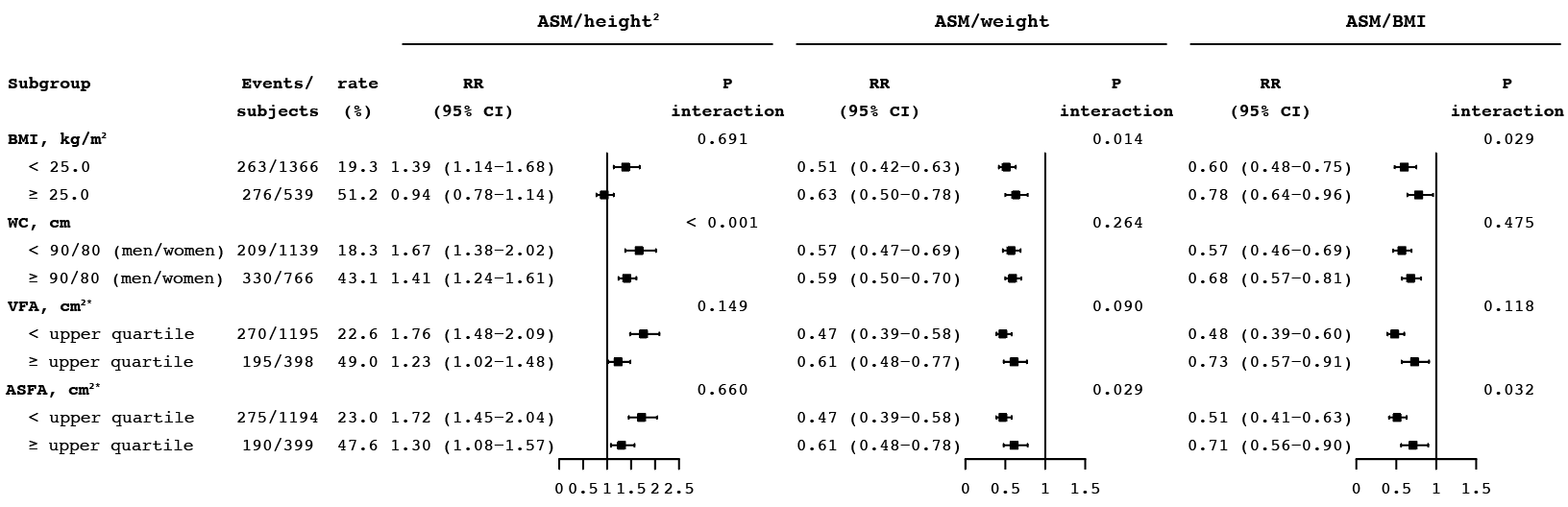


# Figure S4. Associations of ASM/height^2^, ASM/weight, and ASM/BMI (per SD) with incident MASLD stratified by obesity indices.

RR (95% CI) was calculated using the modified Poisson regression model with robust error variance adjusting for sex, age, education attainments, smoking status, drinking status, and leisure-time exercise.

^*^Upper quartile for VFA: men: VFA > 127.0 cm^2^; women: VFA > 116.9 cm^2^; upper quartile for ASFA: men: ASFA > 130.1 cm^2^; women: ASFA > 189.4 cm^2^.

ASM, appendicular skeletal muscle mass; ASFA, abdominal subcutaneous fat area; BMI, body mass index; CI, confidence interval; MASLD, metabolic-dysfunction associated steatosis liver disease; RR, risk ratio; SD, standard deviations; VFA, visceral fat area; WC, waist circumference.

#


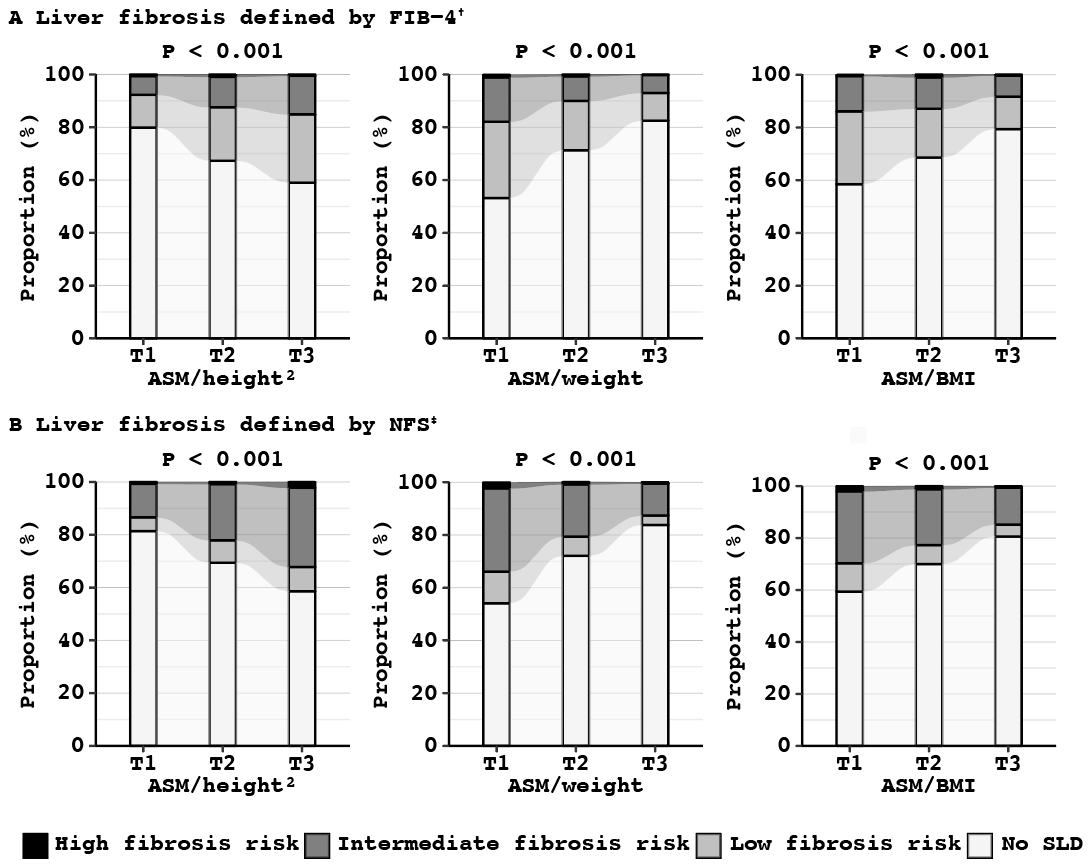


# Figure S5. Proportions of liver fibrosis at follow-up assessed by FIB-4 (A) and NFS (B) stratified by ASM/height^2^, ASM/weight, and ASM/BMI^*^.

P value for trend.

^*^Individuals with high fibrosis risk defined by FIB-4 (n = 287) or NFS (n = 193) were excluded at baseline, respectively.

^†^Low, intermediate, and high fibrosis risk were defined by FIB-4 < 1.3 ( < 65 years) or < 2.0 ( ≥ 65 years old), 1.3 - 2.67 ( < 65 years) or 2.0 - 2.67 ( ≥ 65 years), and > 2.67, respectively.

^‡^Low, intermediate, and high fibrosis risk were defined by NFS < -1.455, -1.455 - 0.676, and > 0.676, respectively.

ASM, appendicular skeletal muscle mass; BMI, body mass index; FIB-4, fibrosis-4; NAFLD, non-alcoholic fatty liver disease; NFS, NAFLD fibrosis score; SLD, steatotic liver disease; T1-T3, tertile 1-tertile 3.


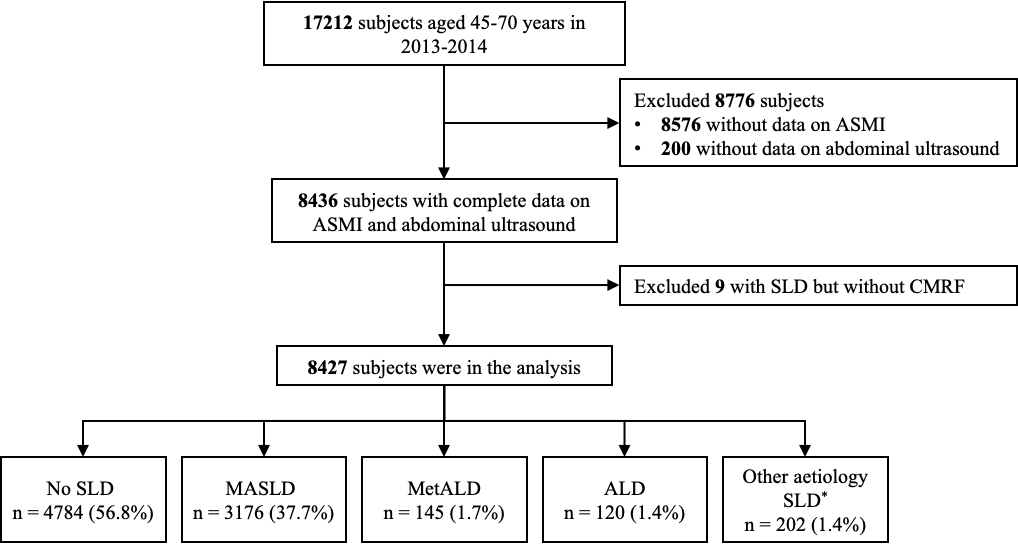


# Figure S6. Flowchart of study population for cross-sectional analysis.

^*^Other aetiology SLD included individuals with positive hepatitis B surface antigen and hepatitis C virus antibody.

ALD, alcohol-associated liver disease; ASMI, appendicular skeletal muscle mass index; CMRF, cardiometabolic risk factors; MASLD, metabolic-dysfunction associated steatosis liver disease; MetALD, metabolic dysfunction and alcohol-associated liver disease; SLD, steatosis liver disease.

# Table S1. Risk models for liver fibrosis.

| **Models** | **Equations** | **Cut-off points for liver fibrosis risk** | | |
| --- | --- | --- | --- | --- |
|  |  | **Low** | **Intermediate** | **High** |
| FIB-4 | age (years) × AST (U/L) /  (platelet count [ × 10^9^/L] × ALT [U/L] ^1/2^) | < 1.3 ( < 65 years) or < 2.0 ( ≥ 65 years) | 1.3 - 2.67 ( < 65 years) or 2.0 - 2.67 ( ≥ 65 years) | > 2.67 |
|  |  |  |  |  |
| NFS | - 1.675 + 0.037 × age (years) + 0.094 × BMI (kg/m^2^) + 1.13 × prediabetes or diabetes (yes = 1, no = 0) + 0.99 × AST/ALT ratio - 0.013 × platelet count ( × 10^9^/L) - 0.66 × ALB (g/dL) | < -1.455 | - 1.455 - 0.676 | > 0.676 |

ALB, albumin; ALT, alanine aminotransferase; AST, aspartate aminotransferase; BMI, body mass index; FIB-4, fibrosis-4; NAFLD, non-alcoholic fatty liver disease; NFS, NAFLD fibrosis score.

# Table S2. Summary of laboratory tests.

| **Item** | **Method** |
| --- | --- |
| FPG | Glucose oxidase method |
| HbA1c | High-performance liquid chromatography |
| FINS | Electrochemiluminescence immunoassay |
| TG | Enzymatic colorimetric method |
| TC | Enzymatic colorimetric method |
| HDL-C | Direct method |
| LDL-C | Direct method |
| ALT | UV-lactate dehydrogenase method |
| AST | UV-Malic dehydrogenase method |
| GGT | γ-glutamyl-p-nitroaniline method |
| UA | Enzymatic method |
| ChE | JSCC transferable method |
| PA | Immune turbidimetric assay |
| Adiponectin | Latex particle enhanced immunoturbidimetric assay |
| FGF21 | Enzyme-linked immunosorbent assay |
| RBP4 | Turbidimetric immunoassay |
| Osteocalcin | Electrochemiluminescence immunoassay |
| HBsAg | Enzyme linked immunosorbent assay |
| HCVAb | Enzyme linked immunosorbent assay |

ALT, alanine aminotransferase; AST, aspartate aminotransferase; ChE, cholinesterase; FGF21, fibroblast growth factor 21; FINS, fasting insulin; FPG, fasting plasma glucose; GGT, gamma-glutamyl transpeptidase; HbA1c, glycated hemoglobin; HBsAg, hepatitis B surface antigen; HCVAb, hepatitis C virus antibody; HDL-C, high-density lipoprotein cholesterol; LDL-C, low-density lipoprotein cholesterol; PA, prealbumin; RBP4, retinol-binding protein-4; TC, total cholesterol; TG, triglyceride; UA, uric acid.

# Table S3. Baseline characteristics of subjects stratified by ASM/height^2*^.

| **Characteristic** | Tertile 1  n = 654 | Tertile 2  n = 656 | Tertile 3  n = 654 | ^†^p value |
| --- | --- | --- | --- | --- |
| **Soci-demographic** |  |  |  |  |
| Age, years | 61.3 (58.4-65.1) | 60.7 (58.0-64.7) | 62.0 (58.8-65.5) | < 0.001 |
| Women, no. (%) | 356 (54.4) | 358 (54.6) | 357 (54.6) | 0.998 |
| Middle school or above, no. (%) | 241 (36.9) | 232 (35.4) | 244 (37.3) | 0.747 |
| **Lifestyles** |  |  |  |  |
| Current smoker, n (%) | 156 (23.9) | 140 (21.3) | 133 (20.3) | 0.284 |
| Current drinker, n (%) | 108 (16.5) | 100 (15.2) | 91 (13.9) | 0.425 |
| Physical activity, n (%) | 30 (4.6) | 39 (5.9) | 24 (3.7) | 0.149 |
| **Clinical** |  |  |  |  |
| SBP, mmHg | 130.0  (120.0-140.8) | 132.0  (122.0-142.0) | 134.0  (124.0-144.4) | < 0.001 |
| DBP, mmHg | 80.0 (75.0-85.0) | 81.0 (77.5-86.1) | 82.0 (78.0-87.5) | < 0.001 |
| FPG, mmol/L | 5.7 (5.4-6.2) | 5.7 (5.4-6.2) | 5.7 (5.3-6.2) | 0.828 |
| HbA1c, % | 5.6 (5.4-5.8) | 5.7 (5.4-5.9) | 5.7 (5.4-6.0) | < 0.001 |
| FINS, μU/mL | 5.0 (3.6-6.9) | 5.8 (4.2-8.0) | 6.1 (4.6-8.2) | < 0.001 |
| HOMA-IR | 1.3 (0.9-1.8) | 1.5 (1.1-2.1) | 1.6 (1.2-2.2) | < 0.001 |
| TG, mmol/L | 1.0 (0.7-1.4) | 1.1 (0.8-1.6) | 1.2 (0.8-1.6) | < 0.001 |
| TC, mmol/L | 5.1 (4.4-5.7) | 5.0 (4.5-5.6) | 4.9 (4.4-5.5) | 0.038 |
| HDL-C, mmol/L | 1.4 (1.2-1.7) | 1.3 (1.1-1.6) | 1.2 (1.1-1.5) | < 0.001 |
| LDL-C, mmol/L | 2.9 (2.4-3.5) | 3.0 (2.5-3.5) | 3.0 (2.5-3.5) | 0.087 |
| ALT, U/L | 14.0 (11.0-18.0) | 15.0 (12.0-20.0) | 15.0 (12.0-20.0) | < 0.001 |
| AST, U/L | 22.0 (19.0-26.0) | 22.0 (19.0-25.0) | 22.0 (19.0-25.0) | 0.693 |
| GGT, U/L | 20.0 (15.0-28.0) | 20.0 (15.0-29.0) | 21.0 (16.0-30.0) | 0.308 |
| UA, μmol/L | 272.5  (229.0-331.8) | 284.0  (242.0-333.0) | 290.0  (243.2-345.0) | 0.014 |
| ChE, U/L | 338.0  (298.0-388.0) | 351.5  (311.8-396.0) | 352.5  (307.0-393.0) | 0.010 |
| PA, mg/L | 268.5  (236.2-304.0) | 268.0  (238.0-305.0) | 264.5  (235.2-298.8) | 0.189 |
| **Anthropometric** |  |  |  |  |
| ASM/height^2^, kg/m^2^ | 6.31 (6.05-7.54) | 6.75 (6.53-8.39) | 7.68 (7.06-9.29) | < 0.001 |
| ASM/weight, % | 30.8 (27.8-34.6) | 30.7 (27.5-35.3) | 31.6 (28.1-36.4) | 0.002 |
| ASM/BMI, m^2^ | 0.79 (0.68-0.95) | 0.77 (0.67-0.98) | 0.79 (0.66-1.01) | 0.701 |
| BMI, kg/m^2^ | 21.6 (20.1-22.9) | 23.7 (22.5-24.9) | 25.6 (24.1-27.1) | < 0.001 |
| WC, cm | 78.0 (73.0-84.0) | 82.0 (78.0-87.0) | 85.0 (80.0-90.0) | < 0.001 |
| VFA, cm^2^ | 78.0 (59.0-102.3) | 100.2 (74.1-120.8) | 109.6 (84.0-140.2) | < 0.001 |
| ASFA, cm^2^ | 105.8  (74.2-142.9) | 129.1  (97.6-168.7) | 143.6  (111.4-181.2) | < 0.001 |
| **Adipokine** |  |  |  |  |
| Adiponectin, μg/mL | 4.5 (3.5-6.2) | 4.4 (3.3-5.8) | 4.3 (3.2-5.6) | 0.003 |
| FGF21, pg/mL | 192.3  (116.9-315.3) | 216.1  (120.1-312.7) | 237.5  (145.5-354.7) | < 0.001 |
| RBP4, mg/L | 45.0 (37.0-54.0) | 45.0 (38.0-56.0) | 44.5 (37.0-55.0) | 0.458 |
| Osteocalcin, ng/mL | 22.6 (17.8-28.6) | 21.4 (16.9-28.1) | 22.2 (17.2-28.2) | 0.172 |

Data were presented as medians (25^th^-75^th^ percentiles) or numbers (proportions) as appropriate.

^*^ASM/height^2^: men: tertile 1, < 8.05; tertile 2, 8.05-< 8.83; tertile 3, ≥ 8.83; women: tertile 1, < 6.36; tertile 2, 6.36-< 6.79; tertile 3, ≥ 6.79.

^†^P value for trend.

ALT, alanine aminotransferase; ASM, appendicular skeletal muscle mass; AST, aspartate aminotransferase; BMI, body mass index; ChE, cholinesterase; DBP, diastolic blood pressure; FGF21, fibroblast growth factor 21; FINS, fasting insulin; FPG, fasting plasma glucose; GGT, gamma-glutamyl transpeptidase; HbA1c, glycated hemoglobin; HDL-C, high-density lipoprotein cholesterol; HOMA-IR, homeostasis model assessment of insulin resistance; LDL-C, low-density lipoprotein cholesterol; PA, prealbumin; RBP4, retinol-binding protein-4; SBP, systolic blood pressure; TC, total cholesterol; TG, triglyceride; UA, uric acid; WC, waist circumference.

# Table S4. Baseline characteristics of subjects stratified by ASM/weight^*^.

| **Characteristic** | Tertile 1  n = 655 | Tertile 2  n = 655 | Tertile 3  n = 654 | ^†^p value |
| --- | --- | --- | --- | --- |
| **Soci-demographic** |  |  |  |  |
| Age, years | 61.0 (58.2-65.0) | 61.8 (58.5-65.2) | 61.4 (58.5-65.1) | 0.364 |
| Women, no. (%) | 357 (54.5) | 357 (54.5) | 357 (54.6) | 0.999 |
| Middle school or above, no. (%) | 239 (36.5) | 223 (34.0) | 255 (39.0) | 0.178 |
| **Lifestyles** |  |  |  |  |
| Current smoker, n (%) | 133 (20.3) | 151 (23.1) | 145 (22.2) | 0.470 |
| Current drinker, n (%) | 106 (16.2) | 103 (15.7) | 90 (13.8) | 0.432 |
| Physical activity, n (%) | 33 (5.0) | 34 (5.2) | 26 (4.0) | 0.530 |
| **Clinical** |  |  |  |  |
| SBP, mmHg | 133.0  (125.0-144.0) | 132.0  (121.0-142.2) | 130.0  (120.0-141.0) | < 0.001 |
| DBP, mmHg | 82.0 (79.0-88.0) | 81.0 (77.0-86.0) | 80.0 (74.0-84.0) | < 0.001 |
| FPG, mmol/L | 5.8 (5.4-6.2) | 5.7 (5.3-6.2) | 5.6 (5.3-6.1) | < 0.001 |
| HbA1c, % | 5.7 (5.4-5.9) | 5.7 (5.4-5.9) | 5.6 (5.3-5.9) | < 0.001 |
| FINS, μU/mL | 6.7 (5.1-9.3) | 5.7 (4.3-7.7) | 4.6 (3.4-6.3) | < 0.001 |
| HOMA-IR | 1.8 (1.3-2.4) | 1.5 (1.1-2.0) | 1.2 (0.8-1.6) | < 0.001 |
| TG, mmol/L | 1.2 (0.9-1.7) | 1.1 (0.8-1.5) | 0.9 (0.7-1.3) | < 0.001 |
| TC, mmol/L | 5.2 (4.6-5.8) | 5.0 (4.4-5.6) | 4.8 (4.3-5.4) | < 0.001 |
| HDL-C, mmol/L | 1.3 (1.1-1.5) | 1.3 (1.1-1.6) | 1.4 (1.1-1.7) | < 0.001 |
| LDL-C, mmol/L | 3.1 (2.7-3.6) | 3.0 (2.6-3.5) | 2.8 (2.3-3.3) | < 0.001 |
| ALT, U/L | 15.0 (12.0-20.0) | 15.0 (12.0-19.0) | 14.0 (11.0-18.0) | < 0.001 |
| AST, U/L | 22.0 (19.0-25.0) | 22.0 (19.0-25.0) | 22.0 (19.0-25.0) | 0.507 |
| GGT, U/L | 22.0 (17.0-31.0) | 21.0 (16.0-31.0) | 18.0 (14.0-25.0) | < 0.001 |
| UA, μmol/L | 296.0  (249.0-350.0) | 285.0  (242.0-339.0) | 268.0  (224.0-320.0) | < 0.001 |
|  |  |  |  |  |
| ChE, U/L | 366.5  (324.2-413.8) | 349.0  (311.0-394.0) | 324.0  (289.0-369.0) | < 0.001 |
| PA, mg/L | 277.0  (246.0-312.0) | 268.0  (241.0-301.0) | 253.5  (227.2-291.0) | < 0.001 |
| **Anthropometric** |  |  |  |  |
| ASM/height^2^, kg/m^2^ | 7.05 (6.45-8.04) | 7.18 (6.48-8.41) | 7.24 (6.53-8.61) | 0.003 |
| ASM/weight, % | 26.8 (25.7-33.4) | 28.8 (27.9-35.6) | 33.4 (30.3-38.1) | < 0.001 |
| ASM/BMI, m^2^ | 0.69 (0.62-0.91) | 0.75 (0.68-0.98) | 0.84 (0.73-1.07) | < 0.001 |
| BMI, kg/m^2^ | 25.1 (23.6-26.6) | 23.6 (22.2-25.0) | 22.0 (20.3-23.6) | < 0.001 |
| WC, cm | 86.0 (81.0-90.0) | 82.0 (77.0-87.0) | 77.0 (72.0-83.0) | < 0.001 |
| VFA, cm^2^ | 112.9 (90.6-136.6) | 94.1 (74.1-119.3) | 73.6 (53.6-98.5) | < 0.001 |
| ASFA, cm^2^ | 152.6  (120.1-194.8) | 129.1  (97.7-167.3) | 104.5  (71.8-130.1) | < 0.001 |
| **Adipokine** |  |  |  |  |
| Adiponectin, μg/mL | 4.2 (3.2-5.6) | 4.2 (3.3-5.5) | 4.9 (3.7-6.5) | < 0.001 |
| FGF21, pg/mL | 222.2  (141.6-346.2) | 208.7  (128.8-328.3) | 211.7  (120.0-323.8) | 0.209 |
| RBP4, mg/L | 48.0 (39.0-58.0) | 45.0 (38.0-55.0) | 43.0 (35.0-51.0) | < 0.001 |
| Osteocalcin, ng/mL | 21.2 (16.8-27.6) | 21.7 (16.9-27.8) | 23.1 (18.3-29.8) | < 0.001 |

Data were presented as medians (25^th^-75^th^ percentiles) or numbers (proportions) as appropriate.

^*^ASM/weight: men: tertile 1, < 34.66; tertile 2, 34.66-< 37.10; tertile 3, ≥ 37.10; women: tertile 1, < 27.07; tertile 2, 27.07-< 29.14; tertile 3, ≥ 29.14.

^†^P value for trend.

ALT, alanine aminotransferase; ASM, appendicular skeletal muscle mass; AST, aspartate aminotransferase; BMI, body mass index; ChE, cholinesterase; DBP, diastolic blood pressure; FGF21, fibroblast growth factor 21; FINS, fasting insulin; FPG, fasting plasma glucose; GGT, gamma-glutamyl transpeptidase; HbA1c, glycated hemoglobin; HDL-C, high-density lipoprotein cholesterol; HOMA-IR, homeostasis model assessment of insulin resistance; LDL-C, low-density lipoprotein cholesterol; PA, prealbumin; RBP4, retinol-binding protein-4; SBP, systolic blood pressure; TC, total cholesterol; TG, triglyceride; UA, uric acid; WC, waist circumference.

# Table S5. Baseline characteristics of subjects stratified by ASM/BMI^*^.

| **Characteristic** | Tertile 1  n = 655 | Tertile 2  n = 655 | Tertile 3  n = 654 | ^†^p value |
| --- | --- | --- | --- | --- |
| **Soci-demographic** |  |  |  |  |
| Age, years | 61.3 (58.4-65.1) | 61.2 (58.4-65.0) | 61.5 (58.4-65.2) | 0.791 |
| Women, no. (%) | 357 (54.5) | 357 (54.5) | 357 (54.6) | 0.999 |
| Middle school or above, no. (%) | 218 (33.3) | 225 (34.4) | 274 (41.9) | 0.002 |
| **Lifestyles** |  |  |  |  |
| Current smoker, n (%) | 145 (22.1) | 150 (22.9) | 134 (20.5) | 0.559 |
| Current drinker, n (%) | 108 (16.5) | 93 (14.2) | 98 (15.0) | 0.503 |
| Physical activity, n (%) | 25 (3.8) | 36 (5.5) | 32 (4.9) | 0.350 |
| **Clinical** |  |  |  |  |
| SBP, mmHg | 133.0  (124.5-143.5) | 132.0  (121.0-142.2) | 131.0  (120.0-141.0) | 0.002 |
| DBP, mmHg | 81.0 (78.0-87.5) | 81.0 (77.0-86.0) | 80.0 (75.6-85.0) | 0.001 |
| FPG, mmol/L | 5.7 (5.4-6.2) | 5.7 (5.3-6.2) | 5.7 (5.3-6.2) | 0.288 |
| HbA1c, % | 5.7 (5.4-5.9) | 5.7 (5.4-5.9) | 5.6 (5.4-5.9) | 0.021 |
| FINS, μU/mL | 6.2 (4.6-8.7) | 5.7 (4.2-7.8) | 5.0 (3.6-6.8) | < 0.001 |
| HOMA-IR | 1.6 (1.2-2.3) | 1.5 (1.0-2.1) | 1.3 (0.9-1.8) | < 0.001 |
| TG, mmol/L | 1.2 (0.8-1.7) | 1.1 (0.8-1.6) | 1.0 (0.7-1.4) | < 0.001 |
| TC, mmol/L | 5.2 (4.6-5.8) | 5.0 (4.4-5.6) | 4.8 (4.3-5.4) | < 0.001 |
| HDL-C, mmol/L | 1.3 (1.1-1.6) | 1.3 (1.1-1.6) | 1.4 (1.1-1.6) | 0.002 |
| LDL-C, mmol/L | 3.1 (2.7-3.6) | 3.0 (2.5-3.5) | 2.8 (2.4-3.3) | < 0.001 |
| ALT, U/L | 15.0 (12.0-20.0) | 14.0 (12.0-19.0) | 14.0 (11.0-19.0) | 0.089 |
| AST, U/L | 22.0 (19.0-25.0) | 22.0 (19.0-25.0) | 22.0 (19.0-25.0) | 0.272 |
| GGT, U/L | 22.0 (16.0-32.0) | 20.0 (15.0-29.0) | 19.0 (14.0-26.0) | < 0.001 |
| UA, μmol/L | 293.0  (245.0-346.0) | 281.0  (240.0-337.5) | 275.0  (226.0-325.0) | < 0.001 |
| ChE, U/L | 362.0  (320.0-414.0) | 348.5  (304.0-391.0) | 329.5  (296.0-375.0) | < 0.001 |
| PA, mg/L | 274.0  (242.0-308.0) | 267.0  (240.0-304.0) | 258.5  (232.0-293.0) | < 0.001 |
| **Anthropometric** |  |  |  |  |
| ASM/height^2^, kg/m^2^ | 7.11 (6.53-8.13) | 7.18 (6.46-8.41) | 7.16 (6.41-8.48) | 0.631 |
| ASM/weight, % | 28.6 (26.0-33.7) | 30.2 (27.8-35.4) | 33.4 (29.9-37.6) | < 0.001 |
| ASM/BMI, m^2^ | 0.64 (0.61-0.89) | 0.70 (0.68-0.99) | 0.84 (0.75-1.09) | < 0.001 |
| BMI, kg/m^2^ | 24.9 (23.3-26.5) | 23.6 (22.2-25.1) | 22.2 (20.4-23.8) | < 0.001 |
| WC, cm | 84.0 (78.0-89.0) | 82.0 (78.0-87.0) | 79.0 (73.2-86.0) | < 0.001 |
| VFA, cm^2^ | 105.4 (80.1-129.4) | 98.3 (75.7-124.5) | 80.3 (58.2-108.5) | < 0.001 |
| ASFA, cm^2^ | 143.1  (103.6-190.2) | 130.5  (98.1-168.1) | 113.4  (79.5-139.6) | < 0.001 |
| **Adipokine** |  |  |  |  |
| Adiponectin, μg/mL | 4.2 (3.3-5.6) | 4.3 (3.3-5.6) | 4.7 (3.6-6.4) | < 0.001 |
| FGF21, pg/mL | 228.5  (144.9-354.1) | 210.5  (126.7-310.8) | 200.4  (119.6-316.5) | 0.002 |
| RBP4, mg/L | 47.0 (38.5-57.0) | 45.0 (37.0-55.0) | 43.0 (37.0-52.0) | 0.001 |
| Osteocalcin, ng/mL | 21.1 (16.7-27.8) | 22.1 (17.5-28.0) | 22.9 (18.0-29.6) | 0.004 |

Data were presented as medians (25^th^-75^th^ percentiles) or numbers (proportions) as appropriate.

^*^ASM/BMI: men: tertile 1, < 0.95; tertile 2, 0.95-< 1.03; tertile 3, ≥ 1.03; women: tertile 1, < 0.65; tertile 2, 0.65-< 0.71; tertile 3, ≥ 0.71.

^†^P value for trend.

ALT, alanine aminotransferase; ASM, appendicular skeletal muscle mass; AST, aspartate aminotransferase; BMI, body mass index; ChE, cholinesterase; DBP, diastolic blood pressure; FGF21, fibroblast growth factor 21; FINS, fasting insulin; FPG, fasting plasma glucose; GGT, gamma-glutamyl transpeptidase; HbA1c, glycated hemoglobin; HDL-C, high-density lipoprotein cholesterol; HOMA-IR, homeostasis model assessment of insulin resistance; LDL-C, low-density lipoprotein cholesterol; PA, prealbumin; RBP4, retinol-binding protein-4; SBP, systolic blood pressure; TC, total cholesterol; TG, triglyceride; UA, uric acid; WC, waist circumference.

# Table S6. Associations of ASM/WC (per SD) with incident SLD and its subtypes after a 4.3-year follow-up.

| Variable | No SLD  (n = 1366) | SLD (n = 598) | | |  | SLD subtypes | | | | | | | |
| --- | --- | --- | --- | --- | --- | --- | --- | --- | --- | --- | --- | --- | --- |
|  |  |  |  |  |  | MASLD (n = 539) | |  | MetALD (n = 38) | |  | ALD (n = 21) | |
|  |  |  | RR, 95% CI | p value |  | RR, 95% CI | p value |  | RR, 95% CI | p value |  | RR, 95% CI | p value |
| Model 1^*^ | Ref. |  | 0.77 (0.68-0.86) | < 0.001 |  | 0.67 (0.56-0.82) | < 0.001 |  | 0.79 (0.48-1.28) | 0.333 |  | 0.56 (0.29-1.09) | 0.088 |
| Model 2^†^ | Ref. |  | 0.77 (0.68-0.87) | < 0.001 |  | 0.66 (0.53-0.82) | < 0.001 |  | 0.65 (0.39-1.09) | 0.102 |  | 0.48 (0.24-0.96) | 0.039 |
| Model 3^‡^ | Ref. |  | 0.77 (0.68-0.87) | < 0.001 |  | 0.67 (0.54-0.83) | < 0.001 |  | 0.67 (0.40-1.12) | 0.127 |  | 0.47 (0.23-0.94) | 0.032 |

RR (95% CI) was calculated using the modified Poisson regression models or multinomial logistic regression models.

^*^Model 1 was adjusted for sex, age, education attainments, smoking status, drinking status, and leisure-time exercise.

^†^Model 2 was adjusted for variables in Model 1 and also for BMI.

^‡^Model 3 was adjusted for variables in Model 2 and also for hypertension, diabetes, TG, and HDL-C.

ALD, alcohol-associated liver disease; ASM, appendicular skeletal muscle mass; BMI, body mass index; CI, confidence interval; HDL-C, high-density lipoprotein cholesterol; MASLD, metabolic-dysfunction associated steatosis liver disease; MetALD, metabolic dysfunction and alcohol-associated liver disease; RR, risk ratio; SD, standard deviations; SLD, steatotic liver disease; TG, triglyceride; WC, waist circumference.

# Table S7. Comparisons of incremental values among ASM/height^2^, ASM/weight, ASM/BMI, and MFR in predicting the incidence of MASLD.

| Variable | AUC  (95% CI) | p value | NRI  (%, 95% CI) | p value | IDI  (%, 95% CI) | p value |
| --- | --- | --- | --- | --- | --- | --- |
| Sex- and- age model | 0.561 (0.533-0.561) | - | ref. | - | ref. | - |
| + ASM/height^2^ | 0.653 (0.626-0.653) | < 0.001 | 42.1 (32.3-51.8) | < 0.001 | 4.5 (3.6-5.5) | < 0.001 |
| + ASM/weight | 0.693 (0.667-0.693) | < 0.001 | 55.6 (46.1-65.1) | < 0.001 | 7.8 (6.6-9.0) | < 0.001 |
| + ASM/BMI | 0.645 (0.618-0.645) | < 0.001 | 34.9 (25.1-44.7) | < 0.001 | 3.9 (3.0-4.7) | < 0.001 |
| + ASM/WC | 0.589 (0.561-0.589) | < 0.001 | 21.1 (11.2-31.0) | < 0.001 | 0.8 (0.4-1.1) | < 0.001 |
| + MFR | 0.730 (0.706-0.730) | < 0.001 | 72.4 (63.5-81.3) | < 0.001 | 9.2 (8.0-10.4) | < 0.001 |

ASM, appendicular skeletal muscle mass; AUC, area under the curve; BMI, body mass index; CI, confidence interval; IDI, integrated discrimination improvement; MASLD, metabolic-dysfunction associated steatosis liver disease; MFR, muscle/fat ratio; NRI, net reclassification index; WC, waist circumference.

# Table S8. Mediation analyses to estimate the indirect, direct, and total effects of ASM/height^2^, ASM/weight, and ASM/BMI on incident MASLD.

| Mediator | ACME^†^  Estimate (95% CI) | ADE^†^  Estimate (95% CI) | Total Effect^†^  Estimate (95% CI) | Percentage Mediated  % (95% CI) | P value^‡^ |
| --- | --- | --- | --- | --- | --- |
| **ASM/height^2^** |  |  |  |  |  |
| SBP, mmHg | 0.007 (0.002 to 0.01) | 0.22 (0.16 to 0.29) | 0.23 (0.17 to 0.30) | 3.2 (0.8 to 6.1) | < 0.001 |
| DBP, mmHg | 0.010 (0.004 to 0.02) | 0.22 (0.15 to 0.29) | 0.23 (0.17 to 0.30) | 4.2 (1.7 to 7.3) | < 0.001 |
| FPG, mmol/L | 0.000 (-0.002 to 0.003) | 0.22 (0.16 to 0.29) | 0.22 (0.17 to 0.30) | 0.2 (-0.9 to 1.4) | 0.768 |
| FINS, μU/mL^*^ | 0.03 (0.02 to 0.05) | 0.18 (0.12 to 0.25) | 0.22 (0.16 to 0.30) | 15.5 (10.8 to 22.4) | < 0.001 |
| HbA1c, % | 0.003 (-0.000 to 0.008) | 0.22 (0.16 to 0.30) | 0.22 (0.16 to 0.30) | 1.4 (-0.0 to 3.4) | 0.066 |
| TC, mmol/L | -0.003 (-0.007 to -0.000) | 0.23 (0.17 to 0.30) | 0.23 (0.17 to 0.30) | -1.1 (-3.1 to -0.1) | 0.038 |
| HDL-C, mmol/L | 0.03 (0.02 to 0.05) | 0.20 (0.14 to 0.26) | 0.24 (0.17 to 0.31) | 13.3 (8.0 to 19.9) | < 0.001 |
| LDL-C, mmol/L | 0.005 (0.001 to 0.010) | 0.22 (0.16 to 0.30) | 0.23 (0.17 to 0.30) | 2.0 (0.3 to 4.4) | 0.024 |
| ALT, U/L | 0.002 (-0.002 to 0.006) | 0.22 (0.16 to 0.30) | 0.23 (0.17 to 0.30) | 1.1 (-0.7 to 2.9) | 0.252 |
| AST, U/L | -0.000 (-0.003 to 0.003) | 0.23 (0.17 to 0.30) | 0.23 (0.17 to 0.30) | -0.1 (-1.2 to 1.4) | 0.898 |
| GGT, U/L | -0.000 (-0.002 to 0.002) | 0.23 (0.17 to 0.30) | 0.23 (0.17 to 0.30) | -0.1 (-0.7 to 0.8) | 0.720 |
| **ASM/weight** |  |  |  |  |  |
| SBP, mmHg | -0.003 (-0.007 to -0.000) | -0.15 (-0.19 to -0.13) | -0.16 (-0.19 to -0.13) | 2.1 (0.0 to 4.7) | 0.048 |
| DBP, mmHg | -0.005 (-0.010 to -0.001) | -0.15 (-0.19 to -0.13) | -0.16 (-0.19 to -0.13) | 3.5 (0.9 to 6.2) | 0.006 |
| FPG, mmol/L | -0.001 (-0.003 to 0.002) | -0.15 (-0.19 to -0.13) | -0.16 (-0.19 to -0.13) | 0.5 (-1.0 to 1.6) | 0.458 |
| FINS, μU/mL^*^ | -0.03 (-0.03 to -0.02) | -0.14 (-0.18 to -0.12) | -0.16 (-0.19 to -0.14) | 15.8 (10.7 to 21.2) | < 0.001 |
| HbA1c, % | -0.001 (-0.003 to 0.001) | -0.15 (-0.19 to -0.13) | -0.15 (-0.19 to -0.13) | 0.6 (-1.0 to 1.7) | 0.410 |
| TC, mmol/L | -0.001 (-0.005 to 0.004) | -0.16 (-0.19 to -0.13) | -0.16 (-0.19 to -0.13) | 0.5 (-2.3 to 3.0) | 0.764 |
| HDL-C, mmol/L | -0.01 (-0.02 to -0.005) | -0.15 (-0.19 to -0.13) | -0.16 (-0.20 to -0.14) | 6.3 (3.2 to 9.9) | < 0.001 |
| LDL-C, mmol/L | -0.006 (-0.01 to -0.001) | -0.16 (-0.19 to -0.13) | -0.16 (-0.19 to -0.13) | 3.6 (0.6 to 6.5) | 0.016 |
| ALT, U/L | -0.001 (-0.003 to 0.001) | -0.16 (-0.19 to -0.13) | -0.16 (-0.19 to -0.13) | 0.8 (-0.5 to 2.2) | 0.222 |
| AST, U/L | -0.000 (-0.002 to 0.001) | -0.16 (-0.19 to -0.13) | -0.16 (-0.19 to -0.13) | 0.1 (-0.7 to 1.1) | 0.822 |
| GGT, U/L | -0.001 (-0.003 to 0.001) | -0.16 (-0.19 to -0.13) | -0.16 (-0.19 to -0.13) | 0.5 (-0.7 to 1.9) | 0.164 |
| **ASM/BMI** |  |  |  |  |  |
| SBP, mmHg | -0.004 (-0.008 to -0.001) | -0.14 (-0.17 to -0.11) | -0.14 (-0.17 to -0.11) | 2.8 (0.7 to 5.6) | 0.006 |
| DBP, mmHg | -0.004 (-0.008 to -0.001) | -0.14 (-0.17 to -0.11) | -0.14 (-0.17 to -0.11) | 2.9 (0.8 to 5.7) | 0.002 |
| FPG, mmol/L | -0.001 (-0.003 to 0.000) | -0.14 (-0.17 to -0.11) | -0.14 (-0.17 to -0.11) | 0.8 (-0.3 to 2.2) | 0.136 |
| FINS, μU/mL^*^ | -0.02 (-0.03 to -0.02) | -0.12 (-0.15 to -0.10) | -0.14 (-0.17 to -0.11) | 15.2 (10.5 to 21.9) | < 0.001 |
| HbA1c, % | -0.002 (-0.004 to 0.000) | -0.14 (-0.16 to -0.11) | -0.14 (-0.16 to -0.11) | 1.2 (-0.1 to 2.7) | 0.090 |
| TC, mmol/L | -0.002 (-0.006 to 0.003) | -0.14 (-0.17 to -0.11) | -0.14 (-0.17 to -0.11) | 1.1 (-2.0 to 4.2) | 0.492 |
| HDL-C, mmol/L | -0.009 (-0.01 to -0.004) | -0.13 (-0.16 to -0.11) | -0.14 (-0.17 to -0.12) | 6.2 (2.7 to 10.6) | < 0.001 |
| LDL-C, mmol/L | -0.007 (-0.01 to -0.002) | -0.14 (-0.17 to -0.11) | -0.14 (-0.17 to -0.11) | 4.6 (1.4 to 8.7) | 0.004 |
| ALT, U/L | -0.001 (-0.003 to 0.000) | -0.14 (-0.17 to -0.11) | -0.14 (-0.17 to -0.11) | 1.1 (-0.2 to 2.5) | 0.114 |
| AST, U/L | 0.000 (-0.001 to 0.003) | -0.14 (-0.17 to -0.11) | -0.14 (-0.17 to -0.11) | -0.3 (-1.9 to 0.9) | 0.614 |
| GGT, U/L | -0.001 (-0.004 to 0.000) | -0.14 (-0.17 to -0.11) | -0.14 (-0.17 to -0.11) | 0.7 (-0.2 to 2.7) | 0.084 |

^*^Log_e_-transformed before analysis.

^†^Two multivariable-adjusted regression models (linear regression model for the mediator and Poisson regression model for the outcome) were established to evaluate these effects. The effects of ASM/height^2^, ASM/weight, and ASM/BMI (per SD) on incident MASLD were adjusted for sex, age, education attainments, smoking status, drinking status, leisure-time exercise. CIs were calculated using percentile bootstrap (replications = 1000).

^‡^P value for percentage mediated.

ACME, average causal mediation effects; ADE, average direct effects; ALT, alanine aminotransferase; ASM, appendicular skeletal muscle mass; AST, aspartate aminotransferase; BMI, body mass index; CI, confidence interval; DBP, diastolic blood pressure; FINS, fasting insulin; FPG, fasting plasma glucose; GGT, gamma-glutamyl transpeptidase; HbA1c, glycated hemoglobin; HDL-C, high-density lipoprotein cholesterol; LDL-C, low-density lipoprotein cholesterol; MASLD, metabolic-dysfunction associated steatosis liver disease; SBP, systolic blood pressure; SD, standard deviations; TC, total cholesterol.

# Table S9. Associations of ASM/height^2^, ASM/weight, and ASM/BMI (per SD) with incident liver fibrosis evaluated by FIB-4 (n = 1660)^*^.

| Variable | No SLD  (n = 1138, 68.6%) |  | SLD with different fibrosis stages | | | | | | | |
| --- | --- | --- | --- | --- | --- | --- | --- | --- | --- | --- |
|  |  |  | Low fibrosis (n = 155, 9.3%)^†^ | |  | Intermediate fibrosis (n = 356, 21.4%)^†^ | |  | High fibrosis (n = 11, 0.7%)^†^ | |
|  |  |  | RR, 95% CI | p value |  | RR, 95% CI | p value |  | RR, 95% CI | p value |
| **ASM/height^2^** |  |  |  |  |  |  |  |  |  |  |
| Model 1^‡^ | Ref. |  | 2.06 (1.67-2.55) | < 0.001 |  | 2.13 (1.61-2.82) | < 0.001 |  | 0.91 (0.36-2.28) | 0.835 |
| Model 2^§^ | Ref. |  | 0.61 (0.45-0.82) | 0.001 |  | 0.63 (0.43-0.93) | 0.019 |  | 0.29 (0.10-0.89) | 0.030 |
| Model 3^¶^ | Ref. |  | 0.61 (0.45-0.82) | 0.001 |  | 0.62 (0.42-0.91) | 0.015 |  | 0.35 (0.11-1.06) | 0.062 |
| **ASM/weight** |  |  |  |  |  |  |  |  |  |  |
| Model 1^‡^ | Ref. |  | 0.31 (0.24-0.40) | < 0.001 |  | 0.31 (0.22-0.43) | < 0.001 |  | 0.22 (0.07-0.68) | 0.008 |
| Model 2^§^ | Ref. |  | 0.59 (0.44-0.79) | < 0.001 |  | 0.61 (0.42-0.89) | 0.010 |  | 0.31 (0.11-0.94) | 0.038 |
| Model 3^¶^ | Ref. |  | 0.59 (0.44-0.79) | 0.001 |  | 0.60 (0.41-0.87) | 0.008 |  | 0.38 (0.13-1.14) | 0.085 |
| **ASM/BMI** |  |  |  |  |  |  |  |  |  |  |
| Model 1^‡^ | Ref. |  | 0.44 (0.34-0.58) | < 0.001 |  | 0.43 (0.30-0.60) | < 0.001 |  | 0.55 (0.17-1.80) | 0.325 |
| Model 2^§^ | Ref. |  | 0.54 (0.41-0.72) | < 0.001 |  | 0.51 (0.36-0.73) | < 0.001 |  | 0.62 (0.19-2.05) | 0.434 |
| Model 3^¶^ | Ref. |  | 0.57 (0.43-0.75) | < 0.001 |  | 0.50 (0.35-0.72) | < 0.001 |  | 0.74 (0.23-2.37) | 0.614 |

RR (95% CI) was calculated using the multinomial logistic regression models.

^*^Individuals with high fibrosis risk at baseline were excluded (n = 287).

^†^Low, intermediate, and high fibrosis risk were defined by FIB-4 < 1.3 ( < 65 years) or < 2.0 ( ≥ 65 years), 1.3 - 2.67 ( < 65 years) or 2.0 - 2.67 ( ≥ 65 years), and > 2.67, respectively.

^‡^Model 1 was adjusted for sex, age, education attainments, smoking status, drinking status, and leisure-time exercise.

^§^Model 2 was adjusted for variables in Model 1 and also for BMI (ASM/height^2^ and ASM/weight) or WC (ASM/BMI).

^¶^Model 3 was adjusted for variables in Model 2 and also for hypertension, diabetes, TG, and HDL-C.

ASM, appendicular skeletal muscle mass; BMI, body mass index; CI, confidence interval; FIB-4, fibrosis-4; HDL-C, high-density lipoprotein cholesterol; MASLD, metabolic-dysfunction associated steatosis liver disease; RR, risk ratio; SD, standard deviations; TG, triglyceride; WC, waist circumference.

# Table S10. Associations of ASM/height^2^, ASM/weight, and ASM/BMI (per SD) with incident liver fibrosis evaluated by NFS (n = 1746)^*^.

| Variable | No SLD  (n = 1223, 70.0%) |  | SLD with different fibrosis stages | | | | | | | |
| --- | --- | --- | --- | --- | --- | --- | --- | --- | --- | --- |
|  |  |  | Low fibrosis (n = 133, 7.6%)^†^ | |  | Intermediate fibrosis (n = 369, 21.0%)^†^ | |  | High fibrosis (n = 21, 1.2%)^†^ | |
|  |  |  | RR, 95% CI | p value |  | RR, 95% CI | p value |  | RR, 95% CI | p value |
| **ASM/height^2^** |  |  |  |  |  |  |  |  |  |  |
| Model 1^‡^ | Ref. |  | 1.87 (1.36-2.58) | < 0.001 |  | 2.40 (1.95-2.96) | < 0.001 |  | 1.88 (0.87-4.07) | 0.109 |
| Model 2^§^ | Ref. |  | 0.55 (0.36-0.85) | 0.006 |  | 0.69 (0.52-0.92) | 0.010 |  | 0.29 (0.11-0.72) | 0.008 |
| Model 3^¶^ | Ref. |  | 0.56 (0.36-0.87) | 0.009 |  | 0.68 (0.51-0.90) | 0.008 |  | 0.31 (0.12-0.78) | 0.012 |
| **ASM/weight** |  |  |  |  |  |  |  |  |  |  |
| Model 1^‡^ | Ref. |  | 0.29 (0.20-0.41) | < 0.001 |  | 0.32 (0.25-0.40) | < 0.001 |  | 0.14 (0.06-0.31) | < 0.001 |
| Model 2^§^ | Ref. |  | 0.53 (0.35-0.80) | 0.002 |  | 0.68 (0.51-0.90) | 0.007 |  | 0.31 (0.12-0.79) | 0.014 |
| Model 3^¶^ | Ref. |  | 0.54 (0.35-0.82) | 0.004 |  | 0.67 (0.51-0.89) | 0.006 |  | 0.34 (0.13-0.86) | 0.023 |
| **ASM/BMI** |  |  |  |  |  |  |  |  |  |  |
| Model 1^‡^ | Ref. |  | 0.37 (0.25-0.55) | < 0.001 |  | 0.45 (0.35-0.58) | < 0.001 |  | 0.23 (0.09-0.60) | 0.003 |
| Model 2^§^ | Ref. |  | 0.46 (0.31-0.70) | < 0.001 |  | 0.56 (0.43-0.73) | < 0.001 |  | 0.36 (0.13-0.97) | 0.044 |
| Model 3^¶^ | Ref. |  | 0.49 (0.33-0.74) | 0.001 |  | 0.57 (0.44-0.74) | < 0.001 |  | 0.38 (0.14-1.01) | 0.053 |

RR (95% CI) was calculated using the multinomial logistic regression models.

^*^Individuals with high fibrosis risk at baseline were excluded (n = 193).

^†^Low, intermediate, and high fibrosis risk were defined by NFS < -1.455, -1.455 - 0.676, and > 0.676, respectively.

^‡^Model 1 was adjusted for sex, age, education attainments, smoking status, drinking status, and leisure-time exercise.

^§^Model 2 was adjusted for variables in Model 1 and also for BMI (ASM/height^2^ and ASM/weight) or WC (ASM/BMI).

^¶^Model 3 was adjusted for variables in Model 2 and also for hypertension, diabetes, TG, and HDL-C.

ASM, appendicular skeletal muscle mass; BMI, body mass index; CI, confidence interval; HDL-C, high-density lipoprotein cholesterol; MASLD, metabolic-dysfunction associated steatosis liver disease; NAFLD, non-alcoholic fatty liver disease; NFS, NAFLD fibrosis score; RR, risk ratio; SD, standard deviations; TG, triglyceride; WC, waist circumference.

# Table S11. Associations of ASM and MFR (per SD) with incident SLD and its subtypes.

| Variable | No SLD  (n = 1366) |  | SLD (n = 598) | |  | SLD subtypes | | | | | | | |
| --- | --- | --- | --- | --- | --- | --- | --- | --- | --- | --- | --- | --- | --- |
|  |  |  |  |  |  | MASLD (n = 539) | |  | MetALD (n = 38) | |  | ALD (n = 21) | |
|  |  |  | RR, 95% CI | p value |  | RR, 95% CI | p value |  | RR, 95% CI | p value |  | RR, 95% CI | p value |
| **ASM** |  |  |  |  |  |  |  |  |  |  |  |  |  |
| Model 1^*^ | Ref. |  | 1.65 (1.46-1.86) | < 0.001 |  | 2.24 (1.82-2.76) | < 0.001 |  | 2.04 (1.27-3.29) | 0.003 |  | 1.18 (0.62-2.26) | 0.619 |
| Model 2^†^ | Ref. |  | 0.92 (0.80-1.05) | 0.200 |  | 0.84 (0.65-1.08) | 0.171 |  | 0.94 (0.52-1.67) | 0.819 |  | 0.56 (0.27-1.19) | 0.133 |
| Model 3^‡^ | Ref. |  | 0.90 (0.79-1.04) | 0.146 |  | 0.84 (0.65-1.08) | 0.166 |  | 0.95 (0.53-1.71) | 0.859 |  | 0.53 (0.24-1.13) | 0.099 |
| **MFR** |  |  |  |  |  |  |  |  |  |  |  |  |  |
| Model 1^*^ | Ref. |  | 0.22 (0.17-0.30) | < 0.001 |  | 0.12 (0.09-0.18) | < 0.001 |  | 0.21 (0.10-0.44) | < 0.001 |  | 0.41 (0.18-0.94) | 0.036 |
| Model 2^†^ | Ref. |  | 0.59 (0.47-0.75) | < 0.001 |  | 0.53 (0.36-0.79) | 0.002 |  | 0.49 (0.21-1.16) | 0.104 |  | 0.85 (0.32-2.27) | 0.752 |
| Model 3^‡^ | Ref. |  | 0.61 (0.48-0.78) | < 0.001 |  | 0.56 (0.37-0.84) | 0.005 |  | 0.53 (0.22-1.28) | 0.160 |  | 0.92 (0.32-2.61) | 0.868 |

RR (95% CI) was calculated using the modified Poisson regression models or multinomial logistic regression models.

^*^Model 1 was adjusted for sex, age, education attainments, smoking status, drinking status, and leisure-time exercise.

^†^Model 2 was adjusted for variables in Model 1 and also for BMI.

^‡^Model 3 was adjusted for variables in Model 2 and also for hypertension, diabetes, TG, and HDL-C.

ALD, alcohol-associated liver disease; ASM, appendicular skeletal muscle mass; BMI, body mass index; CI, confidence interval; HDL-C, high-density lipoprotein cholesterol; MASLD, metabolic-dysfunction associated steatosis liver disease; MetALD, metabolic dysfunction and alcohol-associated liver disease; MFR, muscle/fat ratio; RR, risk ratio; SD, standard deviations; SLD, steatotic liver disease; TG, triglyceride.

# Table S12. Associations of ASM/height^2^, ASM/weight, and ASM/BMI (per SD) with incident lean MASLD^*^ or non-lean MASLD.

| Variable | No MASLD  (n = 1366, 71.9%) |  | Lean MASLD (n = 63, 3.3%) | |  | Non-lean MASLD (n = 472, 24.8%) | |
| --- | --- | --- | --- | --- | --- | --- | --- |
|  |  |  | RR, 95% CI | p value |  | RR, 95% CI | p value |
| **ASM/height^2^** |  |  |  |  |  |  |  |
| Model 1^†^ | Ref. |  | 0.43 (0.28-0.67) | < 0.001 |  | 3.02 (2.47-3.69) | < 0.001 |
| Model 2^‡^ | Ref. |  | 0.38 (0.22-0.69) | 0.001 |  | 0.68 (0.52-0.89) | 0.006 |
| Model 3^§^ | Ref. |  | 0.38 (0.21-0.68) | 0.001 |  | 0.67 (0.51-0.89) | 0.005 |
| **ASM/weight** |  |  |  |  |  |  |  |
| Model 1^†^ | Ref. |  | 0.56 (0.34-0.93) | 0.023 |  | 0.25 (0.20-0.31) | < 0.001 |
| Model 2^‡^ | Ref. |  | 0.37 (0.22-0.61) | < 0.001 |  | 0.65 (0.49-0.86) | 0.002 |
| Model 3^§^ | Ref. |  | 0.36 (0.22-0.61) | < 0.001 |  | 0.65 (0.49-0.86) | 0.002 |
| **ASM/BMI** |  |  |  |  |  |  |  |
| Model 1^†^ | Ref. |  | 0.65 (0.38-1.09) | 0.102 |  | 0.35 (0.28-0.45) | < 0.001 |
| Model 2^‡^ | Ref. |  | 0.66 (0.39-1.12) | 0.121 |  | 0.47 (0.36-0.60) | < 0.001 |
| Model 3^§^ | Ref. |  | 0.70 (0.41-1.19) | 0.185 |  | 0.48 (0.37-0.62) | < 0.001 |

RR (95% CI) was calculated using the multinomial logistic regression models.

^*^Lean MASLD was defined as individuals with MASLD in addition to BMI < 23.0 kg/m^2^.

^†^Model 1 was adjusted for sex, age, education attainments, smoking status, drinking status, and leisure-time exercise.

^‡^Model 2 was adjusted for variables in Model 1 and also for BMI (ASM/height^2^ and ASM/weight) or WC (ASM/BMI).

^§^Model 3 was adjusted for variables in Model 2 and also for hypertension, diabetes, TG, and HDL-C.

ASM, appendicular skeletal muscle mass; BMI, body mass index; CI, confidence interval; FIB-4, fibrosis-4; HDL-C, high-density lipoprotein cholesterol; MASLD, metabolic-dysfunction associated steatosis liver disease; RR, risk ratio; SD, standard deviations; TG, triglyceride; WC, waist circumference.

# Table S13. Associations between baseline characteristics (per SD) and incident MASLD.

| Variable | Numbers | Model 1^†^  (RR, 95% CI) | p value | Model 2^‡^  (RR, 95% CI) | p value | Model 3^§^  (RR, 95% CI) | p value |
| --- | --- | --- | --- | --- | --- | --- | --- |
| HOMA-IR^*^ | 1904 | 1.32 (1.25-1.40) | < 0.001 | 1.14 (1.07-1.22) | < 0.001 | 1.13 (1.05-1.22) | 0.001 |
| TG, mmol/L^*^ | 1905 | 1.25 (1.17-1.33) | < 0.001 | 1.13 (1.05-1.20) | < 0.001 | 1.16 (1.00-1.34) | 0.044 |
| UA, μmol/L^*^ | 1904 | 1.27 (1.17-1.37) | < 0.001 | 1.14 (1.05-1.23) | 0.001 | 1.12 (1.03-1.21) | 0.007 |
| ChE, U/L^*^ | 1903 | 1.25 (1.17-1.33) | < 0.001 | 1.15 (1.08-1.23) | < 0.001 | 1.14 (1.06-1.22) | < 0.001 |
| PA, mg/L^*^ | 1905 | 1.24 (1.15-1.33) | < 0.001 | 1.18 (1.09-1.27) | < 0.001 | 1.18 (1.09-1.28) | < 0.001 |
| Adiponectin, μg/mL | 1713 | 0.75 (0.68-0.83) | < 0.001 | 0.81 (0.74-0.89) | < 0.001 | 0.83 (0.75-0.91) | < 0.001 |
| RBP4, mg/L^*^ | 1905 | 1.25 (1.16-1.34) | < 0.001 | 1.19 (1.11-1.28) | < 0.001 | 1.21 (1.13-1.30) | < 0.001 |
| Osteocalcin, ng/mL^*^ | 1905 | 0.87 (0.80-0.94) | < 0.001 | 0.93 (0.86-0.99) | 0.032 | 0.92 (0.86-0.99) | 0.031 |
| FGF21, pg/mL^*^ | 1690 | 1.15 (1.04-1.28) | 0.010 | 1.02 (0.93-1.13) | 0.644 | 0.99 (0.91-1.09) | 0.877 |

RR (95% CI) was calculated using the modified Poisson regression model with robust error variance.

^*^Log_e_-transformed before analysis.

^†^Model 1 was adjusted for sex, age, education attainments, smoking status, drinking status, and leisure-time exercise.

^‡^Model 2 was adjusted for variables in Model 1 and also for BMI.

^§^Model 3 was adjusted for variables in Model 2 and also for hypertension, diabetes, TG, and HDL-C.

BMI, body mass index; ChE, cholinesterase; CI, confidence interval; FGF21, fibroblast growth factor 21; HOMA-IR, homeostasis model assessment of insulin resistance; MASLD, metabolic-dysfunction associated steatosis liver disease; PA, prealbumin; RBP4, retinol-binding protein-4; RR, risk ratio; SD, standard deviations; TG, triglyceride; UA, uric acid.

# Table S14. Associations of ASM/height^2^, ASM/weight, and ASM/BMI (per SD) with prevalent SLD and its subtypes based on cross-sectional data (n = 8427).

| Variable | No SLD  (n = 4784) |  | SLD  (n = 3643) | |  | SLD subtypes | | | | | | | | | | |
| --- | --- | --- | --- | --- | --- | --- | --- | --- | --- | --- | --- | --- | --- | --- | --- | --- |
|  |  |  |  |  |  | MASLD  (n = 3176) | |  | MetALD  (n = 145) | |  | ALD  (n = 120) | |  | Other aetiology SLD^*^  (n = 202) | |
|  |  |  | OR, 95% CI | p value |  | OR, 95% CI | p value |  | OR, 95% CI | p value |  | OR, 95% CI | p value |  | OR, 95% CI | p value |
| **ASM/height^2^** |  |  |  |  |  |  |  |  |  |  |  |  |  |  |  |  |
| Model 1^†^ | Ref. |  | 3.55  (3.26-3.86) | < 0.001 |  | 3.52  (3.22-3.85) | < 0.001 |  | 3.35  (2.61-4.29) | < 0.001 |  | 4.21  (3.21-5.52) | < 0.001 |  | 3.70  (2.93-4.68) | < 0.001 |
| Model 2^‡^ | Ref. |  | 0.47  (0.41-0.53) | < 0.001 |  | 0.46  (0.40-0.53) | < 0.001 |  | 0.42  (0.27-0.63) | < 0.001 |  | 0.55  (0.35-0.86) | 0.009 |  | 0.50  (0.35-0.70) | < 0.001 |
| Model 3^§^ | Ref. |  | 0.49  (0.42-0.56) | < 0.001 |  | 0.48  (0.41-0.55) | < 0.001 |  | 0.48  (0.31-0.73) | 0.001 |  | 0.65  (0.41-1.03) | 0.065 |  | 0.50  (0.35-0.71) | < 0.001 |
| **ASM/weight** |  |  |  |  |  |  |  |  |  |  |  |  |  |  |  |  |
| Model 1^†^ | Ref. |  | 0.15  (0.13-0.16) | < 0.001 |  | 0.14  (0.13-0.16) | < 0.001 |  | 0.17  (0.12-0.25) | < 0.001 |  | 0.22  (0.14-0.33) | < 0.001 |  | 0.15  (0.11-0.20) | < 0.001 |
| Model 2^‡^ | Ref. |  | 0.44  (0.39-0.50) | < 0.001 |  | 0.44  (0.38-0.50) | < 0.001 |  | 0.42  (0.28-0.63) | < 0.001 |  | 0.59  (0.38-0.92) | 0.021 |  | 0.47  (0.33-0.67) | < 0.001 |
| Model 3^§^ | Ref. |  | 0.46  (0.41-0.53) | < 0.001 |  | 0.45  (0.39-0.52) | < 0.001 |  | 0.49  (0.32-0.74) | 0.001 |  | 0.71  (0.45-1.11) | 0.133 |  | 0.47  (0.32-0.68) | < 0.001 |
| **ASM/BMI** |  |  |  |  |  |  |  |  |  |  |  |  |  |  |  |  |
| Model 1^†^ | Ref. |  | 0.27  (0.24-0.30) | < 0.001 |  | 0.25  (0.23-0.28) | < 0.001 |  | 0.39  (0.29-0.54) | < 0.001 |  | 0.51  (0.36-0.72) | < 0.001 |  | 0.28  (0.20-0.39) | < 0.001 |
| Model 2^‡^ | Ref. |  | 0.41  (0.36-0.46) | < 0.001 |  | 0.39  (0.35-0.44) | < 0.001 |  | 0.48  (0.34-0.67) | < 0.001 |  | 0.64  (0.44-0.92) | 0.017 |  | 0.44  (0.31-0.61) | < 0.001 |
| Model 3^§^ | Ref. |  | 0.44  (0.39-0.50) | < 0.001 |  | 0.42  (0.37-0.48) | < 0.001 |  | 0.55  (0.39-0.78) | 0.001 |  | 0.73  (0.50-1.06) | 0.097 |  | 0.45  (0.32-0.64) | < 0.001 |

OR (95% CI) was calculated using the binary logistic regression models and multinomial logistic regression models.

^*^Other aetiology SLD included individuals with positive hepatitis B surface antigen and hepatitis C virus antibody.

^†^Model 1 was adjusted for sex, age, education attainments, smoking status, drinking status, and leisure-time exercise.

^‡^Model 2 was adjusted for variables in Model 1 and also for BMI (ASM/height^2^ and ASM/weight) or WC (ASM/BMI).

^§^Model 3 was adjusted for variables in Model 2 and also for hypertension, diabetes, TG, and HDL-C.

ALD, alcohol-associated liver disease; ASM, appendicular skeletal muscle mass; BMI, body mass index; CI, confidence interval; HDL-C, high-density lipoprotein cholesterol; MASLD, metabolic-dysfunction associated steatosis liver disease; MetALD, metabolic dysfunction and alcohol-associated liver disease; OR, odds ratio; SD, standard deviations; SLD, steatotic liver disease; TG, triglyceride; WC, waist circumference.

# Table S15. Associations of ASM/height^2^, ASM/weight, and ASM/BMI (per SD) with incident SLD and its subtypes after imputing missing data on body composition.^*^

| Variable | No SLD  (n = 2630) |  | SLD (n = 1003) | |  | SLD subtypes | | | | | | | |
| --- | --- | --- | --- | --- | --- | --- | --- | --- | --- | --- | --- | --- | --- |
|  |  |  |  |  |  | MASLD (n = 905) | |  | MetALD (n = 55) | |  | ALD (n = 43) | |
|  |  |  | RR, 95% CI | p value |  | RR, 95% CI | p value |  | RR, 95% CI | p value |  | RR, 95% CI | p value |
| **ASM/height^2^** |  |  |  |  |  |  |  |  |  |  |  |  |  |
| Model 1^†^ | Ref. |  | 1.65 (1.51-1.81) | < 0.001 |  | 2.22 (1.88-2.62) | < 0.001 |  | 1.87 (1.20-2.93) | 0.006 |  | 1.41 (0.83-2.38) | 0.202 |
| Model 2^‡^ | Ref. |  | 0.81 (0.73-0.90) | < 0.001 |  | 0.69 (0.57-0.85) | < 0.001 |  | 0.63 (0.37-1.05) | 0.076 |  | 0.53 (0.29-0.95) | 0.034 |
| Model 3^§^ | Ref. |  | 0.80 (0.72-0.89) | < 0.001 |  | 0.70 (0.57-0.85) | < 0.001 |  | 0.65 (0.38-1.09) | 0.102 |  | 0.56 (0.30-1.03) | 0.063 |
| **ASM/weight** |  |  |  |  |  |  |  |  |  |  |  |  |  |
| Model 1^†^ | Ref. |  | 0.48 (0.42-0.55) | < 0.001 |  | 0.29 (0.24-0.35) | < 0.001 |  | 0.28 (0.16-0.51) | < 0.001 |  | 0.44 (0.22-0.89) | 0.023 |
| Model 2^‡^ | Ref. |  | 0.79 (0.70-0.90) | < 0.001 |  | 0.65 (0.52-0.82) | < 0.001 |  | 0.48 (0.26-0.86) | 0.013 |  | 0.69 (0.34-1.40) | 0.297 |
| Model 3^§^ | Ref. |  | 0.79 (0.70-0.89) | < 0.001 |  | 0.66 (0.53-0.83) | < 0.001 |  | 0.48 (0.27-0.87) | 0.015 |  | 0.70 (0.34-1.45) | 0.334 |
| **ASM/BMI** |  |  |  |  |  |  |  |  |  |  |  |  |  |
| Model 1^†^ | Ref. |  | 0.54 (0.47-0.61) | < 0.001 |  | 0.38 (0.31-0.47) | < 0.001 |  | 0.60 (0.33-1.08) | 0.086 |  | 0.46 (0.24-0.88) | 0.019 |
| Model 2^‡^ | Ref. |  | 0.67 (0.59-0.76) | < 0.001 |  | 0.51 (0.41-0.63) | < 0.001 |  | 0.65 (0.36-1.21) | 0.173 |  | 0.50 (0.26-0.95) | 0.036 |
| Model 3^§^ | Ref. |  | 0.68 (0.60-0.77) | < 0.001 |  | 0.53 (0.43-0.67) | < 0.001 |  | 0.67 (0.36-1.25) | 0.209 |  | 0.51 (0.26-1.00) | 0.050 |

RR (95% CI) was calculated using the modified Poisson regression models or multinomial logistic regression models.

^*^ASM/height^2^, ASM/weight, and ASM/BMI were imputed using a random forest algorithm based on age and sex.

^†^Model 1 was adjusted for sex, age, education attainments, smoking status, drinking status, and leisure-time exercise.

^‡^Model 2 was adjusted for variables in Model 1 and also for BMI (ASM/height^2^ and ASM/weight) or WC (ASM/BMI).

^§^Model 3 was adjusted for variables in Model 2 and also for hypertension, diabetes, TG, and HDL-C.

ALD, alcohol-associated liver disease; ASM, appendicular skeletal muscle mass; BMI, body mass index; CI, confidence interval; HDL-C, high-density lipoprotein cholesterol; MASLD, metabolic-dysfunction associated steatosis liver disease; MetALD, metabolic dysfunction and alcohol-associated liver disease; RR, risk ratio; SD, standard deviations; SLD, steatotic liver disease; TG, triglyceride; WC, waist circumference.

# Reference

S1. Chen P, Hou X, Hu G, Wei L, Jiao L, Wang H, et al. Abdominal subcutaneous adipose tissue: a favorable adipose depot for diabetes? Cardiovasc Diabetol 2018;17:93.

S2. Liang Y, Chen H, Liu Y, Hou X, Wei L, Bao Y, et al. Association of MAFLD With Diabetes, Chronic Kidney Disease, and Cardiovascular Disease: A 4.6-Year Cohort Study in China. J Clin Endocrinol Metab 2022;107:88-97.

S3. Farrell GC, Chitturi S, Lau GK, Sollano JD, Asia-Pacific Working Party on N. Guidelines for the assessment and management of non-alcoholic fatty liver disease in the Asia-Pacific region: executive summary. J Gastroenterol Hepatol 2007;22:775-777.

S4. Sterling RK, Lissen E, Clumeck N, Sola R, Correa MC, Montaner J, et al. Development of a simple noninvasive index to predict significant fibrosis in patients with HIV/HCV coinfection. Hepatology 2006;43:1317-1325.

S5. Angulo P, Hui JM, Marchesini G, Bugianesi E, George J, Farrell GC, et al. The NAFLD fibrosis score: a noninvasive system that identifies liver fibrosis in patients with NAFLD. Hepatology 2007;45:846-854.

S6. Rose GA, Blackburn H. Cardiovascular survey methods. Monogr Ser World Health Organ 1968;56:1-188.

S7. Unger T, Borghi C, Charchar F, Khan NA, Poulter NR, Prabhakaran D, et al. 2020 International Society of Hypertension Global Hypertension Practice Guidelines. Hypertension 2020;75:1334-1357.

S8. American Diabetes Association Professional Practice Committee. 2. Classification and Diagnosis of Diabetes: Standards of Medical Care in Diabetes-2022. Diabetes Care 2022;45:S17-S38.

S9. Matthews DR, Hosker JP, Rudenski AS, Naylor BA, Treacher DF, Turner RC. Homeostasis model assessment: insulin resistance and beta-cell function from fasting plasma glucose and insulin concentrations in man. Diabetologia 1985;28:412-419.

S10. Tingley D, Teppei H, Mit Y, Keele L, Imai K. Mediation: R Package for Causal Mediation Analysis. J Stat Softw 2014;59.

S11. Schwenzer NF, Springer F, Schraml C, Stefan N, Machann J, Schick F. Non-invasive assessment and quantification of liver steatosis by ultrasound, computed tomography and magnetic resonance. J Hepatol 2009;51:433-445.

S12. Chang Y, Ryu S, Kim Y, Cho YK, Sung E, Kim HN, et al. Low Levels of Alcohol Consumption, Obesity, and Development of Fatty Liver With and Without Evidence of Advanced Fibrosis. Hepatology 2020;71:861-873.

S13. Hernaez R, Lazo M, Bonekamp S, Kamel I, Brancati FL, Guallar E, et al. Diagnostic accuracy and reliability of ultrasonography for the detection of fatty liver: a meta-analysis. Hepatology 2011;54:1082-1090.

S14. McPherson S, Stewart SF, Henderson E, Burt AD, Day CP. Simple non-invasive fibrosis scoring systems can reliably exclude advanced fibrosis in patients with non-alcoholic fatty liver disease. Gut 2010;59:1265-1269.

S15. Shah AG, Lydecker A, Murray K, Tetri BN, Contos MJ, Sanyal AJ. Comparison of noninvasive markers of fibrosis in patients with nonalcoholic fatty liver disease. Clin Gastroenterol Hepatol 2009;7:1104-1112.
